# Ligand-dependent downregulation of MR1 cell surface expression

**DOI:** 10.1073/pnas.2003136117

**Published:** 2020-04-27

**Authors:** Mariolina Salio, Wael Awad, Natacha Veerapen, Claudia Gonzalez-Lopez, Corinna Kulicke, Dominic Waithe, Anne W. J. Martens, David M. Lewinsohn, Judith V. Hobrath, Liam R. Cox, Jamie Rossjohn, Gurdyal S. Besra, Vincenzo Cerundolo

**Affiliations:** ^a^MRC Human Immunology Unit, MRC Weatherall Institute of Molecular Medicine, University of Oxford, Oxford OX3 9DS, United Kingdom;; ^b^Infection and Immunity Program, Biomedicine Discovery Institute, Monash University, Clayton, VIC 3800, Australia;; ^c^Department of Biochemistry and Molecular Biology, Monash University, Clayton, VIC 3800, Australia;; ^d^Institute of Microbiology and Infection, School of Biosciences, University of Birmingham, Edgbaston B15 2TT, Birmingham, United Kingdom;; ^e^Division of Pulmonary and Critical Care Medicine, Department of Medicine, Oregon Health & Science University, Portland, OR 97239;; ^f^Research Department, Portland Veterans Administration Healthcare System, Portland, OR 97239;; ^g^MRC Centre for Computational Biology, The Wolfson Imaging Centre, MRC Weatherall Institute of Molecular Medicine, University of Oxford, Oxford OX3 9DS, United Kingdom;; ^h^Drug Discovery Unit, College of Life Sciences, University of Dundee, Dundee DD1 5EH, United Kingdom;; ^i^School of Chemistry, University of Birmingham, Birmingham B15 2TT, United Kingdom;; ^j^Australian Research Council Centre of Excellence in Advanced Molecular Imaging, Monash University, Clayton, VIC 3800, Australia;; ^k^Institute of Infection and Immunity, Cardiff University School of Medicine, Heath Park, Cardiff CF14 4XN, United Kingdom

## Abstract

The antigen-presenting molecule MR1 presents riboflavin-based metabolites to Mucosal-Associated Invariant T (MAIT) cells. While MR1 egress to the cell surface is ligand-dependent, the ability of small-molecule ligands to impact on MR1 cellular trafficking remains unknown. Arising from an in silico screen of the MR1 ligand-binding pocket, we identify one ligand, 3-([2,6-dioxo-1,2,3,6-tetrahydropyrimidin-4-yl]formamido)propanoic acid, DB28, as well as an analog, methyl 3-([2,6-dioxo-1,2,3,6-tetrahydropyrimidin-4-yl]formamido)propanoate, NV18.1, that down-regulate MR1 from the cell surface and retain MR1 molecules in the endoplasmic reticulum (ER) in an immature form. DB28 and NV18.1 compete with the known MR1 ligands, 5-OP-RU and acetyl-6-FP, for MR1 binding and inhibit MR1-dependent MAIT cell activation. Crystal structures of the MAIT T cell receptor (TCR) complexed with MR1-DB28 and MR1-NV18.1, show that these two ligands reside within the A′-pocket of MR1. Neither ligand forms a Schiff base with MR1 molecules; both are nevertheless sequestered by a network of hydrophobic and polar contacts. Accordingly, we define a class of compounds that inhibits MR1 cellular trafficking.

## Significance

MR1 is a monomorphic major histocompatibility complex (MHC) class I-like molecule that presents ligands to Mucosal Associated Invariant T cells. MR1 antigen presentation at the cell surface is tightly regulated by ligand availability. Although previously described MR1 ligands facilitate translocation of ER-resident MR1 to the cell surface, we describe nonmicrobial ligands, DB28 and its ester analogue NV18.1, which retain MR1 in the ER in an immature ligand-receptive form and competitively inhibit stimulatory ligands. We provide the molecular and functional basis underpinning the interactions of this class of ligands with MR1.

Mucosal-Associated Invariant T (MAIT) cells are a subset of evolutionarily conserved nonmajor histocompatibility complex (MHC)-restricted T cells, which are very abundant in human mucosal tissues, in peripheral blood, and in the liver (1, 2). Similar to type I NKT cells, human MAIT cells express a semi-invariant T cell receptor (TCR) composed of the Vα7.2 chain rearranged mainly to Jα33 and paired with a limited number of Vβ chains, mostly TRBV6, TRBV13, and TRBV20 (3, 4). MAIT cells recognize small microbial metabolites presented by the monomorphic MHC class I-related molecule, MR1 (1, 2). The physiological roles of MAIT cells remain unclear, but they are known to be involved in protective immunity (2, 5–7), possibly through modulation of innate and adaptive immune responses (8, 9). Moreover, the role of MAIT cells in cancer (10) and inflammatory diseases, such as obesity (11), diabetes (12), multiple sclerosis (13), and inflammatory bowel disease (14), has been highlighted, and recent reports have suggested they may also play a role in tissue repair (15, 16). Activation of MAIT cells induces the production of various proinflammatory cytokines, predominantly IFN-γ, TNF-α, IL-2, and IL-17 (17, 18), and their potent cytolytic activity allows them to kill infected cells (19).

Unlike MHC molecules, MR1 does not constitutively present antigens, but is found in the endoplasmic reticulum (ER) of all cells in a ligand-receptive conformation (20). The potency of known MAIT cell agonists appears to correlate with their ability to form a Schiff base with MR1 Lys43 located within the A′-pocket, thus allowing MR1 to egress to the cell surface, where the presence of a ribityl moiety in the covalently bound agonist allows for an interaction with the MAIT TCR (21–23). To date, the strongest MAIT cell agonists are 5-(2-oxopropylideneamino)-6-_D_-ribitylaminouracil (5-OP-RU) and 5-(2-oxoethylideneamino)-6-_D_-ribitylaminouracil (5-OE-RU), both pyrimidine-based intermediates along the riboflavin biosynthetic pathway (24). Several bacterial and fungal species synthesize riboflavin (23), and MAIT cells have been shown to possess MR1-dependent antimicrobial activity against infected antigen-presenting cells (5, 6). Conversely, vitamin B9 metabolites [including the folic acid derivative 6-formylpterin, 6-FP and its acetylated derivative Ac-6-FP (23, 25)] are strong MR1 binders and induce MR1 expression at the cell surface; however, the resulting complexes do not activate MAIT cells because they lack the ribityl moiety (22). Drug and drug-like molecules (including diclofenac and salicylates) also bind MR1 and either weakly activate or inhibit MAIT cells (26). However, it remains unknown whether there are other ligands that impact MR1-dependent antigen presentation.

Through an in silico screen, we have identified additional MR1-binding ligands. We describe a ligand that down-regulates MR1 cell-surface expression and provide a molecular basis for its interactions with MR1.

## Results

### Identification of Nonmicrobial MAIT Cell Agonists.

**Fig. 1.**
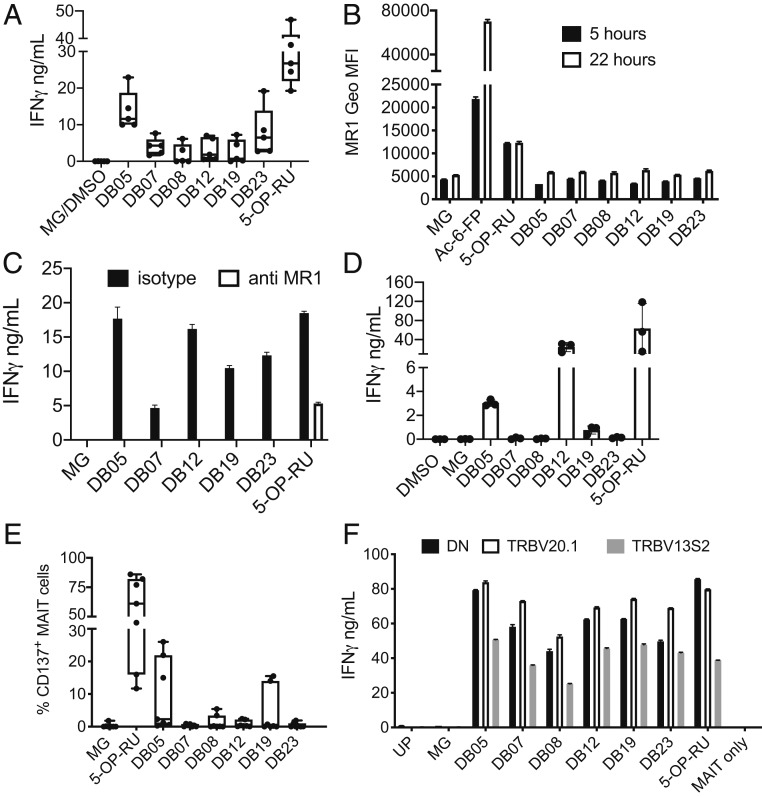
MAIT cell stimulation by the DB series of agonists. (*A*) THP1-MR1 cells were pulsed with the indicated compounds (100 μM MG, 20 μg/mL DB compounds, 50 ng/mL 5-OP-RU) and incubated with MAIT cells. IFN-γ was measured in the supernatant after 36 h of coculture. Box and whiskers bars, minimum to maximum, with all points indicated; *n* = 5. (*B*) The DB series of MAIT cell agonists do not induce MR1 up-regulation. THP1-MR1 cells were pulsed 5 or 22 h with 20 μg/mL of the indicated compounds, 100 μM MG, 1 μg/mL Ac-6-FP, or 1 μg/mL 5-OP-RU. Depicted is the cell surface expression of MR1 at 5 h (black bars) or 22 h (white bars) measured by FACS. Data are mean ± SD of technical duplicates. One experiment is representative of two. (*C*) MR1-dependent presentation of the DB series of agonists. THP1-MR1 cells were pulsed with the indicated compounds (100 μM MG, 20 μg/mL for DB compounds, 50 ng/mL for 5-OP-RU) and incubated with MAIT cells in the presence of isotype control or blocking anti-MR1 antibodies. IFN-γ was measured in the supernatant after 36 h of coculture. Data are mean ± SD of technical duplicates. One experiment is representative of two. (*D*) DC were pulsed with the indicated compounds (100 μM MG, 20 μg/mL DB compounds, 50 ng/mL 5-OP-RU) and incubated with MAIT cells. IFN-γ was measured in the supernatant after 36 h of coculture. Average from technical duplicates from three different donors. One experiment is representative of two. (*E*) Ex vivo MAIT cell activation by the DB series of agonists. Whole blood was stimulated overnight with the indicated compounds. MAIT cell activation, depicted as percentage of CD137 expression, was assessed by flow cytometry. Box and whiskers bars, minimum to maximum, with all points indicated; *n* = 7. (*F*) TCRβ chain expression influences reactivity to DB MAIT cell agonists. MAIT cells expressing TRBV20.1, TRBV13S2, and neither of those two chains (DN) were sorted from a single donor and incubated with THP1-MR1 pulsed with the indicated compounds (100 μM MG, 20 μg/mL for DB compounds, 50 ng/mL for 5-OP-RU). UP, unpulsed. IFN-γ was measured in the supernatant after 36 h of coculture. Data are mean ± SD of technical duplicates. One experiment is representative of two.

To identify MR1 binding ligands, we performed in silico screening using the crystal structures of the MAIT TCR in complex with MR1–antigen complexes [PDB codes 4L4V and 4LCC (22, 27)]. A total of 44,022 compounds were selected for docking runs, based on searches for fragment size substructures s1-s20 (*SI Appendix*, Fig. S1 and *Supplementary Methods*). Compound selection and constraints imposed during docking are detailed in the supplementary methods. Using this strategy, 80 commercial compounds were selected as potential MR1 ligands, of which 52 compounds were pulsed on MR1 overexpressing cells, alongside the canonical MAIT cell ligand 5-OP-RU, synthesized and validated in house (*SI Appendix*, Fig. S2 *A* and *B*). MAIT cell stimulatory activity was observed when THP1-MR1 cells (Fig. 1*A* and *SI Appendix*, Fig. S4*A*) were pulsed with compounds DB5, DB7, DB8, DB12, DB15, DB19, and DB23, whose chemical structures are shown in *SI Appendix*, Fig. S3. Overall, these compounds were three to nine times less potent than 5-OP-RU (*SI Appendix*, Fig. S4*A*). Unlike Ac-6-FP and 5-OP-RU (26), none of the tested compounds induced detectable up-regulation of cell-surface MR1, neither after 5 nor 22 h (Fig. 1*B*). Presentation was MR1-dependent, as determined using the blocking anti-MR1 26.5 monoclonal antibody (28) (Fig. 1*C*) and pulsing the compounds on MR1-KO THP1 cells (*SI Appendix*, Fig. S4*B*). Consistent with their weaker potency, presentation by THP1 cells required a higher level of MR1 expression (THP1-MR1 WT), whereas THP1 cells nonoverexpressing MR1 were unable to present any of the DB compounds (*SI Appendix*, Fig. S4*B*). In addition, presentation was reduced or abrogated when THP1 cells expressing GPI-linked molecules were used (*SI Appendix*, Fig. S4*B*), suggesting internalization and possibly endo-lysosomal loading is required. However, we were unable to detect any MAIT cell activation by fixed THP1-MR1, even after pulsing with the potent agonist 5-OP-RU; therefore, we did not investigate intracellular trafficking further. Compounds DB5, DB12, and DB19 were also presented by monocyte-derived dendritic cells (Fig. 1*D*). In this experimental setting, MAIT cell activation was also MR1-dependent (*SI Appendix*, Fig. S4*C*). We next tested the compounds on unfractionated cells in whole blood and identified MAIT cells by Vα7.2 and CD161 co-staining. MAIT cell activation (measured by CD137 up-regulation) with compounds DB7, DB8, DB12, and DB19 was observed in some, but not all, of the seven donors tested (Fig. 1*E*); this may reflect pairing of different ΤCR β-chains with the canonical MAIT TCR α-chain (3). Indeed, in one donor, we observed a lower response by MAIT cells expressing the TRBV13S2 chain as compared with the TRBV20.1 chain or neither of those two chains (Fig. 1*F*). We also confirmed TCR-mediated recognition of some of the DB compounds using Jurkat cells transduced with a MAIT TCR composed of the canonical TCR α-chain paired with TRBV20.1 or 6.4 (*SI Appendix*, Fig. S4 *D* and *E*) (29). In conclusion, we have defined a series of compounds that bind MR1 and can activate, through MR1-TCR interaction, MAIT cells expressing a variety of TCR β chains.

### DB28 Down-Regulates Cell Surface Expression of MR1.

**Fig. 2.**
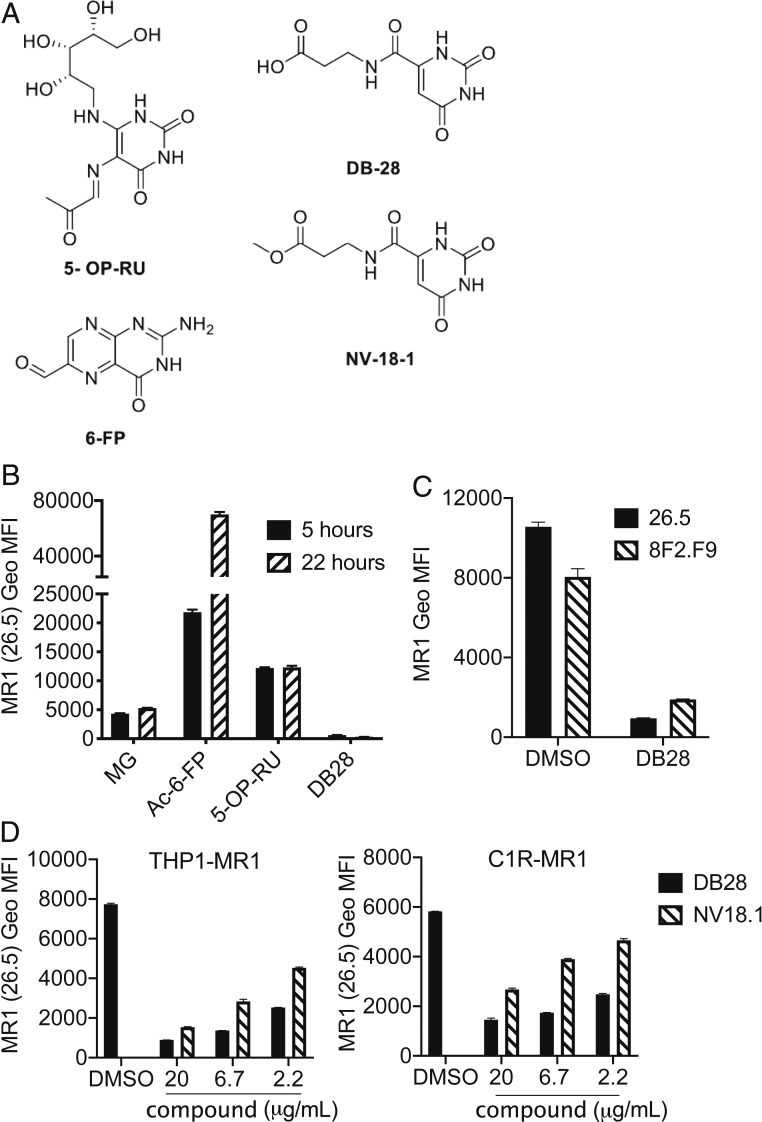
Characterization of DB28. (*A*) Chemical structures of the MR1 ligands used in this study: 5-OP-RU, 6-FP, DB28, and NV18.1. (*B*–*D*) DB28 and NV18.1 down-regulate MR1 from the cell surface. (*B*) THP1-MR1 cells were pulsed for 5 or 22 h with the indicated ligands (MG 50 μM, Ac-6-FP 1 μg/mL, 5-OP-RU 5 μg/mL, DB28 20 μg/mL) before staining with anti-MR1 (26.5) antibody. (*C*) THP1-MR1 cells pulsed overnight with DMSO or DB28 (20 μg/mL) were stained with the two indicated anti-MR1 antibodies. (*D*) THP1-MR1 (*Left*) or C1R-MR1 (*Right*) were pulsed overnight with the indicated concentrations of DB28 or NV18.1 before staining with anti-MR1 (26.5) antibody. Geo MFI ± SD of technical duplicates are plotted in each graph. Data representative of three experimental replicates.

When testing the 52 compounds for ligand-induced MR1 up-regulation, we noticed that, unlike Ac-6-FP and 5-OP-RU, which potently up-regulate MR1 cell-surface expression (24, 25), compound DB28 (3-[(2,6-dioxo-1,2,3,6-tetrahydropyrimidin-4-yl)formamido]propanoic acid, Fig. 2*A*) not only failed to up-regulate MR1 at the cell surface of THP1-MR1 cells but also reduced its expression to almost background levels of staining, using the anti-MR1 monoclonal antibody 26.5 (Fig. 2*B*). The structural formula of compound DB28, and its ester derivative NV18.1 (methyl 3-[(2,6-dioxo-1,2,3,6-tetrahydropyrimidin-4-yl)formamido]propanoate), are shown in Fig. 2*A*. 5-OP-RU and Ac-6-FP covalently bind within the MR1 A′-pocket by forming a Schiff base with MR1 Lys43 (24, 25), which triggers MR1 egress from the ER and trafficking to the cell surface (20). DB28 has a terminal carboxylic acid, while the NV18.1 is its methyl ester analog; thus, without undergoing reduction, neither is able to form a Schiff base with MR1. Cell-surface MR1 down-regulation by DB28 and NV18.1 was observed with two different conformation-specific MR1 antibodies, 26.5 and 8F2.F9 (30, 31) (Fig. 2*C*), where NV18.1 was less potent than DB28 (Fig. 2*D*). MR1 down-regulation was observed when the compounds were tested in both myeloid cells (THP1-MR1) and EBV-transformed B cells (C1R-MR1) (Fig. 2*D*). The effect was specific for MR1, as no down-regulation of MHC-I or CD1d molecules was observed at the surface of THP1 cells (*SI Appendix*, Fig. S5).

**Fig. 3.**
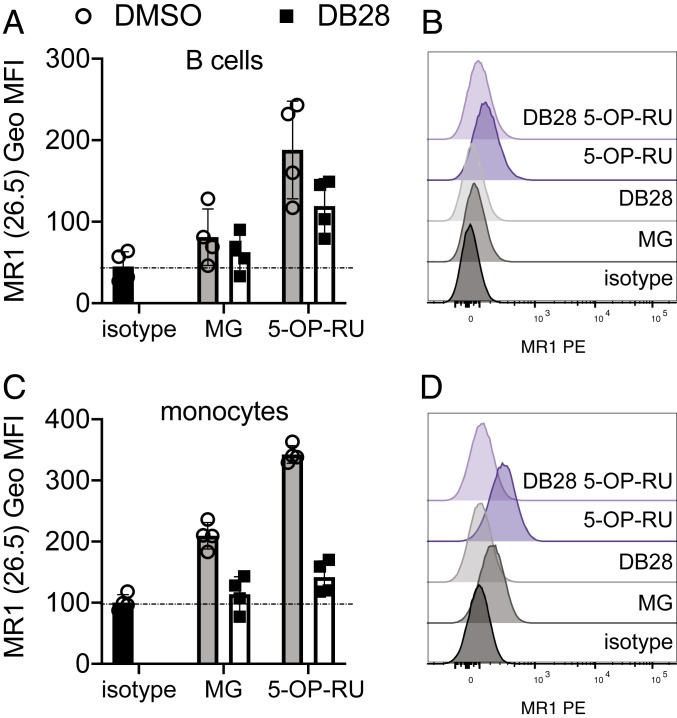
DB28 down-regulates MR1 from the cell surface of primary cells. CD2-depleted PBMC were incubated overnight with MG (50 μM), 5-OP-RU (1 μg/mL) with or without DB28 (20 μg/mL), and MR1 expression on the surface of gated live B cells (*A* and *B*) or monocytes (*C* and *D*) was determined by flow cytometry. Cumulative data of geometrical mean fluorescence intensity of four different blood donors (*A* and *C*); representative FACS histograms (*B* and *D*). The background staining of isotype control is shown in black.

The MR1 transcript is ubiquitous, but expression on primary cells is low (32, 33). Nevertheless, we observed down-regulation of basal and 5-OP-RU-induced surface expression of MR1 by DB28 in primary B cells and monocytes freshly isolated from four healthy donors (Fig. 3), confirming results previously obtained with THP1 cells overexpressing MR1.

**Fig. 4.**
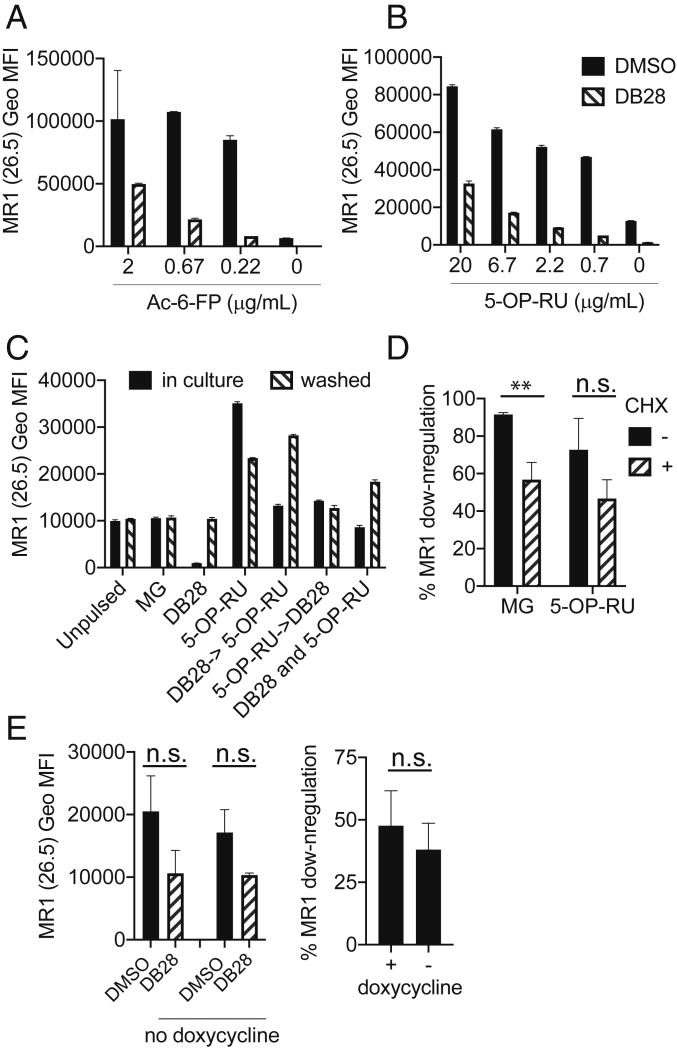
DB28 competes with 5-OP-RU for MR1 binding. (*A* and *B*) THP1-MR1 cells were pulsed with 20 μg/mL DB28 for 2 h before addition of the indicated concentrations of Ac-6-FP (*A*) or 5-OP-RU (*B*). Cells were stained after 18 h. (*C*) THP1-MR1 cells were pulsed with 20 μg/mL DB28 2 h before, concurrently, or 2 h after addition of 5-OP-RU (5 μg/mL). The compounds were kept in culture for 7 h or were washed after 2 h, and cells were chased for 5 h before staining with anti-MR1 (26.5) antibody. Geo MFI ± SD of technical duplicates are plotted in each graph. Data (*A*–*C*) are representative of three experimental replicates. (*D*) THP1-MR1 cells were pulsed with 20 μg/mL DB28 and MG or 5-OP-RU in the presence (hatched bars) or absence (black bars) of 10 μM cycloheximide (CHX) to block protein synthesis. Plotted is the percentage of MR1 down-regulation for each experimental condition (*n* = 4 experimental replicates for MG, 3 for 5-OP-RU; multiple *t* test, **P ≤ 0.005). (*E*) MR1-GFP expression was induced in BEAS2B cells with doxycycline; after 16 h, cells were pulsed with DB28 (20 μg/mL) for 8 h in the presence or absence of doxycycline. Geo MFI ± SD of surface MR1 expression (*Left*) or percentage of MR1 down-regulation (*Right*) are plotted. *n* = 5 experimental replicates with doxycycline, 3 without (*t* test, n.s).

We next tested whether DB28 inhibits the up-regulation of MR1 induced by other ligands. As shown in Fig. 4 *A* and *B*, DB28 abrogated Ac-6-FP- or 5-OP-RU-induced up-regulation of MR1 surface expression, and this effect was stronger when DB28 was in molar excess, suggesting competition for MR1 binding. We added DB28 either concurrently with, 2 h before, or 2 h after 5-OP-RU. In all cases, DB28 reduced MR1 cell surface expression. This effect was reversible, since it persisted as long as DB28 was kept in culture for the duration of the assay and not washed away after the first 5 h (Fig. 4*C*). The reversibility of the DB28 effect prompted us to investigate the contribution of protein synthesis, which is not required for ligand-induced up-regulation of MR1 surface expression (20). At steady state, DB28 down-regulated surface expression of 90% of MR1 molecules, whereas only 60% of MR1 molecules were down-regulated in the presence of the protein synthesis inhibitor cycloheximide. Likewise, in the presence of 5-OP-RU, DB28 down-regulated 70% of MR1 molecules, but only 50% when cells were also treated with cycloheximide (although this difference did not reach statistical significance, Fig. 4*D*). To avoid potential off-target effects of cycloheximide, we tested the epithelial cell line BEAS2B expressing a tetracycline-inducible MR1 construct tagged with GFP (34, 35). Upon doxycycline treatment, more than 90% of cells became GFP positive and 10% to 15% of cells expressed cell surface MR1 (*SI Appendix*, Fig. S6). There was a trend toward lower MR1 down-regulation in the absence of doxycycline; however, it did not reach statistical significance (Fig. 4*E*). In conclusion, we observed selective down-regulation of MR1 cell surface expression by DB28 and NV18.1, which was only marginally affected by blocking protein synthesis.

### Requirements for DB28 Down-Modulation of MR1 Expression.

**Fig. 5.**
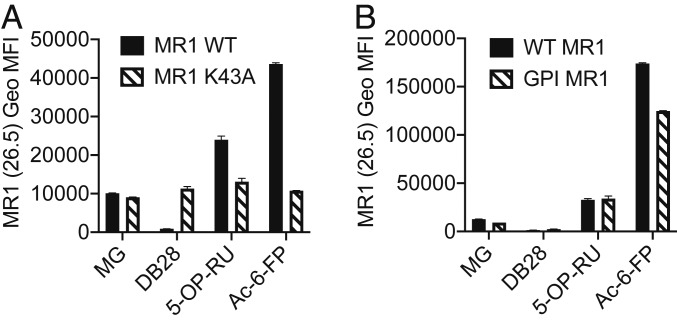
DB28 down-regulates GPI-linked MR1 molecules, but not K43A mutants. THP1 MR1 KO cells overexpressing WT MR1 molecules, K43A mutants (*A*), or GPI-linked MR1 (*B*) were pulsed for 5 h with the indicated ligands (MG 50 μM, Ac-6-FP 1 μg/mL, 5-OP-RU 5 μg/mL, DB28 20 μg/mL) before staining with anti-MR1 (26.5) antibody. Geo MFI ± SD of technical duplicates are plotted in each graph. Data representative of three experimental replicates.

As previously shown (20), the Lys43Ala mutation facilitates the release of MR1 molecules from the ER even in the absence of vitamin B metabolites. To dissect the molecular mechanism by which DB28 induces MR1 down-regulation, we generated THP1 cells expressing Lys43Ala-mutated MR1 molecules (*SI Appendix*, Fig. S7) (20). For these experiments, we used MR1 KO THP1 cells (36) to avoid residual activity of WT MR1 molecules on Lys43Ala MR1-bound ligands. MR1-Lys43Ala molecules were insensitive to DB28-induced modulation, suggesting intracellular retention rather than down-regulation from the cell surface as the main mechanism for DB28-dependent MR1 down-regulation (Fig. 5*A*). To investigate the role of the transmembrane and cytoplasmic domains of MR1 in DB28 modulation of MR1 expression, we transduced THP1 MR1 KO cells with lentiviral particles encoding for GPI-linked MR1 molecules (*SI Appendix*, Fig. S7). Cell surface expression of GPI-linked MR1 molecules was reduced in the presence of DB28, suggesting that the transmembrane and cytoplasmic domains are not required for the observed effect (Fig. 5*B*).

### DB28 Retains MR1 in the ER in an Immature Form.

**Fig. 6.**
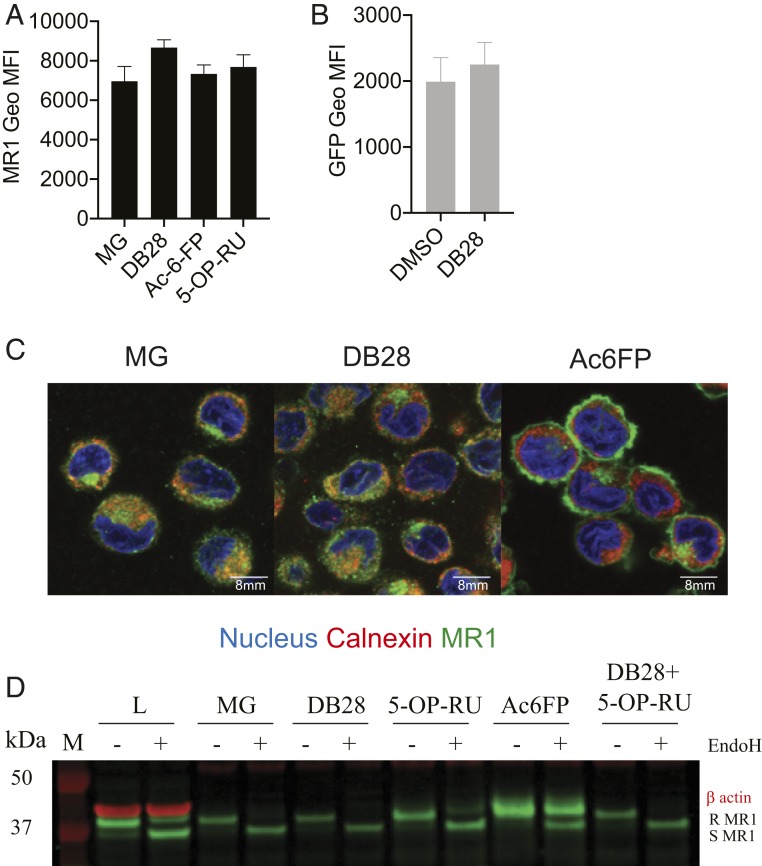
DB28 retains MR1 molecules in the ER/Golgi in an EndoH-sensitive conformation. (*A*) THP1-MR1 cells were pulsed overnight with the indicated ligands (MG 50 μM, Ac-6-FP 1 μg/mL, 5-OP-RU 5 μg/mL, DB28 20 μg/mL) before staining with a polyclonal anti-MR1 antibody. (*B*) BEAS2B expressing MR1-GFP tagged were pulsed overnight with DB28 (20 μg/mL). Plotted is the Geo MFI ± SD of GFP expression of technical duplicates. One experiment is representative of two. (*C*) Confocal images of THP1-MR1 cells pulsed overnight with MG 50 μM, Ac-6-FP 1 μg/mL, or DB28 20 μg/mL. Cells were stained with DAPI (blue), anti-calnexin (red), and anti-MR1 26.5 (green). Images were processed as described in the *Methods*. (Scale bar, 8 μm.) Images are representative of one experiment out of five; cumulative Pearson correlation coefficient is shown in *SI Appendix*, Fig. S8. (*D*) THP1-MR1 cells were pulsed overnight with the indicated ligands (MG 50 μM, Ac-6-FP 1 μg/mL, 5-OP-RU 5 μg/mL, DB28 20 μg/mL), and MR1 molecules were immunoprecipitated with 26.5 antibody. Shown is the analysis of EndoH treated (+) or not (–) MR1 molecules (green) from lysates (L) or the indicated immunoprecipitates. β-actin (red) was determined in the lysate as the loading control. Samples were processed as described in the *Methods*. R, EndoH-resistant; S, EndoH-sensitive. One experiment is representative of three.

To investigate the fate of MR1 on incubation of cells with the ligand DB28, we fixed and permeabilized the cells to determine total MR1 content by flow cytometry. Staining with a polyclonal anti-MR1 antibody revealed the total MR1 content was unaffected, thus ruling out degradation of MR1 molecules (Fig. 6*A*); this was further confirmed with the epithelial cell line, BEAS2B, expressing a tetracycline-inducible MR1 construct tagged with GFP (34, 35) or constitutively expressing GFP-tagged MR1 molecules. In both cell lines, in the presence of DB28, we observed down-regulation of MR1 from the cell surface (Fig. 4*E* and *SI Appendix*, Fig. S6*B*, respectively), but the total GFP content remained unaffected (Fig. 6*B* and *SI Appendix*, Fig. S6*C*, respectively). Consistent with these findings, when we sampled the intracellular distribution of MR1 molecules by confocal microscopy, in the presence of vehicle, MR1 molecules were preferentially colocalized within the ER and Golgi compartments, as previously reported, while they translocated to the cell surface with Ac-6-FP (20) (Fig. 6*C* and *SI Appendix*, Fig. S8*A*). In contrast, in the presence of DB28, they remained in the ER/Golgi compartments. Furthermore, MR1 molecules immunoprecipitated with 26.5 antibody [which recognizes folded MR1 molecules (28)] remained EndoH sensitive in the presence of DB28, as expected from their ER localization. In the presence of 5-OP-RU, they acquired partial EndoH resistance, which was abrogated by coincubation with DB28 (Fig. 6*D* and *SI Appendix*, Fig. S8*B*). In conclusion, DB28 retains the immature form of MR1 within the ER.

### DB28 Prevents MAIT Cell Activation by Agonist Ligands.

**Fig. 7.**
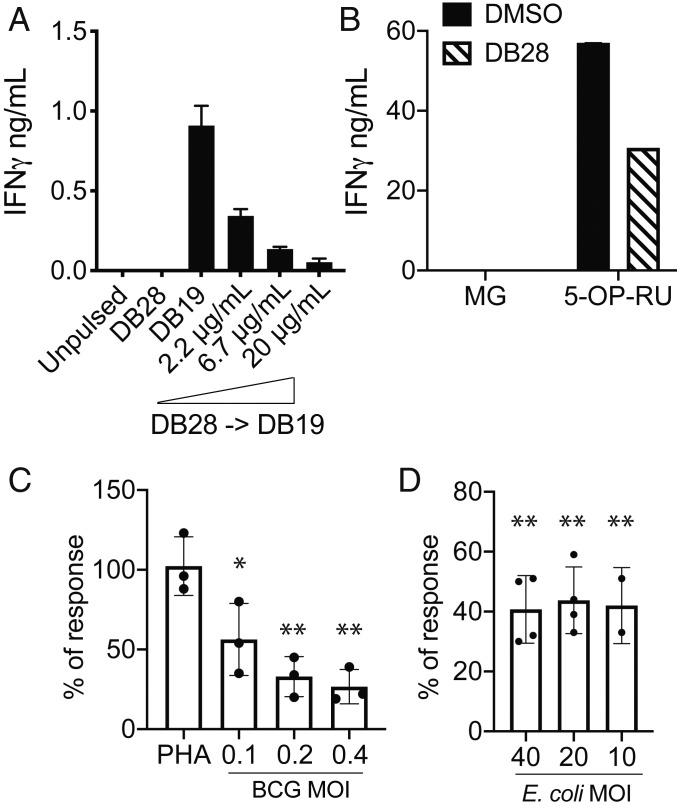
DB28 reduces the stimulatory activity of agonist-pulsed MR1-expressing antigen-presenting cells. (*A* and *B*) THP1-MR1 were pulsed with the indicated concentrations of DB28 2 h before pulsing with DB19 (20 μg/mL) (*A*) or 5-OP-RU (5 ng/mL) (B) and addition of MAIT cells. In *B*, DB28 was used at 20 μg/mL. IFN-γ (mean ± SD of technical duplicates) was measured in the supernatant collected after 16 h of stimulation. (*C*) MAIT cell stimulation by BEAS2B infected with bacillus Calmette-Guérin (BCG) at the indicated MOI, in the presence or absence of DB28 (20 μg/mL). Plotted is the percentage of response DB28/DMSO, where DMSO controls have been normalized to 100. *n* = 3 experimental replicates, each performed in technical duplicates (raw data for one donor shown in *SI Appendix*, Fig. S9*B*). Multiple *t* test, *P ≤ 0.05; **P ≤ 0.005. (*D*) MAIT cell stimulation in PBMC infected with the indicated MOI of *E. coli* in the presence or absence of DB28 (20 μg/mL). Plotted is the percentage of response DB28/DMSO. *n* = 4 experimental replicates (2 for MOI 10). (Raw data for one donor shown in *SI Appendix*, Fig. S9 *C* and *D*.) Multiple *t* test, **P ≤ 0.005.

Upon recognition of their cognate antigen via the TCR, MAIT cells release cytokines in an MR1-dependent manner (6, 19). In agreement with a lack of effect of DB28 on CD1d expression (*SI Appendix*, Fig. S5), iNKT cell activation was unaffected when THP1 cells were pulsed with αGalCer and DB28 (*SI Appendix*, Fig. S9*A*). Consistent with the ability of DB28 to down-regulate MR1 cell surface expression even in the presence of MAIT cell agonists (Fig. 4*B*), we observed dose-dependent inhibition of MAIT cell activation when DB28 was pulsed on THP1-MR1 cells with an agonist ligand (Fig. 7 *A* and *B*). Likewise, presentation of vitamin B metabolites by epithelial cells infected with bacillus Calmette-Guérin (Fig. 7*C* and *SI Appendix*, Fig. S9*B*) or by peripheral blood mononuclear cells (PBMC) infected with *Escherichia coli* (Fig. 7*D* and *SI Appendix*, Fig. S9*C*) was significantly reduced in the presence of DB28. This effect was specific to MR1-dependent stimulation, as activation of MAIT cells with phytohemagglutinin was not affected by DB28 (Fig. 7*C* and *SI Appendix*, Fig. S9*B*), nor was activation of bystander CD161^neg^ T cells upon bacterial exposure (*SI Appendix*, Fig. S9*D*). We confirmed that even in bacillus Calmette-Guérin-infected BEAS2B epithelial cells (*SI Appendix*, Fig. S10 *A* and *B*) and *E. coli*-infected myeloid cells (THP1; *SI Appendix*, Fig. S10 *C* and *D*), DB28 down-regulated cell surface MR1 (*SI Appendix*, Fig. S10 *A* and *C*), but not total MR1 (*SI Appendix*, Fig. S10 *B* and *D*).

MR1 molecules are highly evolutionarily conserved, with 90% sequence homology between the α1 and α2 domains of humans and mice (32). In vitro experiments with murine bone marrow-derived DC confirmed the ability of DB28 to inhibit MR1-dependent human MAIT cell activation (*SI Appendix*, Fig. S11*A*). We next injected i.v. DB28 to assess its effect on 5-OP-RU-dependent in vivo MAIT cell activation (*SI Appendix*, Fig. S11*B*). Despite a 9 to 90 range of molar excess of DB28, we did not observe inhibition of MAIT cell activation, which we hypothesize might be a consequence of the rapid clearance of the compound, as it does not form a Schiff base with MR1 molecules.

In conclusion, these results demonstrate that, in vitro, DB28 acts as a competitive inhibitor for MAIT cell-activating ligands.

### MR1-DB28 and MR1-NV18.1 Display Very Weak Binding to MAIT TCRs.

**Fig. 8.**
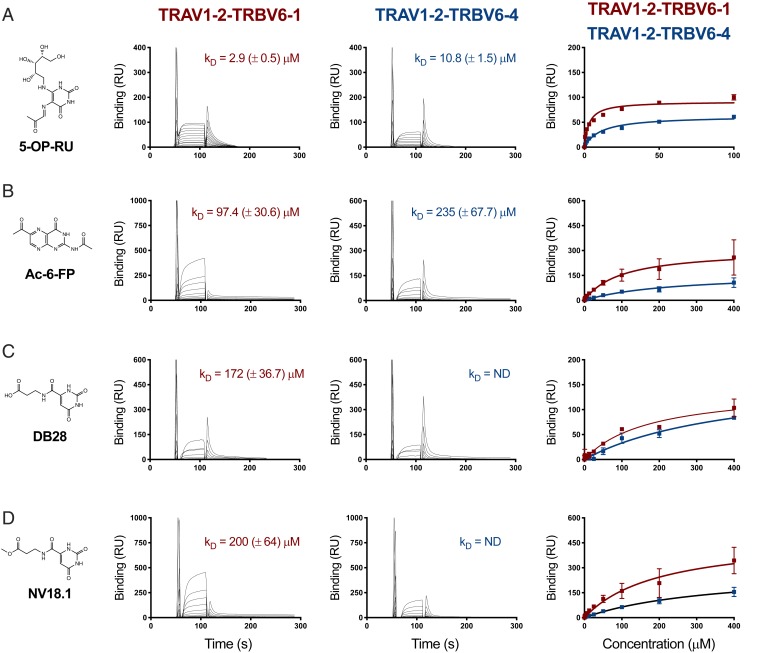
Steady-state affinity measurements of MR1-Ag and MAIT TCRs. (*A*) SPR affinity measurements for MR1 presenting (*A*) 5-OP-RU, (*B*) Ac-6-FP, (*C*) DB28, and (*D*) NV18.1 compounds against two different MAIT TCRs: A-F7 (TRAV1-2-TRBV6-1) and TRAV1-2-TRBV6-4. SPR runs were conducted in duplicate, three independent experiments. The SPR sensorgrams (*Middle*), equilibrium curves (*Right*), and steady state *K*_D_ values (µM) were prepared in Prism 7.

We next measured the binding affinity of MR1 loaded with 5-OP-RU, Ac-6-FP, DB28, and NV18.1 ligands toward two MAIT TCRs (A-F7 [TRAV1-2-TRBV6-1] and #6 [TRAV1-2-TRBV6-4] TCRs) (25), using surface plasmon resonance (SPR; Fig. 8). As previously reported (25), the 5-OP-RU agonist exhibited affinities to MAIT TCRs ranging from ∼3 to 10 μM, whereas the folate antagonist Ac-6-FP showed weak binding (97.4 ± 30.6 and 235 ± 67.7 μM to A-F7 and TRBV6-4 TCRs, respectively) (24, 25). Consistent with the absence of the ribityl tail and the lack of MAIT cell activation, both DB28 and NV-18.1 revealed extremely low binding to AF-7 TCR (*K*_D_ = 172.0 ± 36.7 and 200.0 ± 64.0 μM, respectively), while we could not measure binding to TRBV6-4 TCR (*K*_D_ = ND). Collectively, even if the MR1 complexes with inhibitors DB28 and NV18.1 made it to the cell surface, they would exhibit very weak affinities to MAIT TCRs, in agreement with their inability to stimulate MAIT cells.

### Crystal Structures of MR1-DB28 and MR1-NV18.1 Complexes Bound to MAIT TCR.

**Fig. 9.**
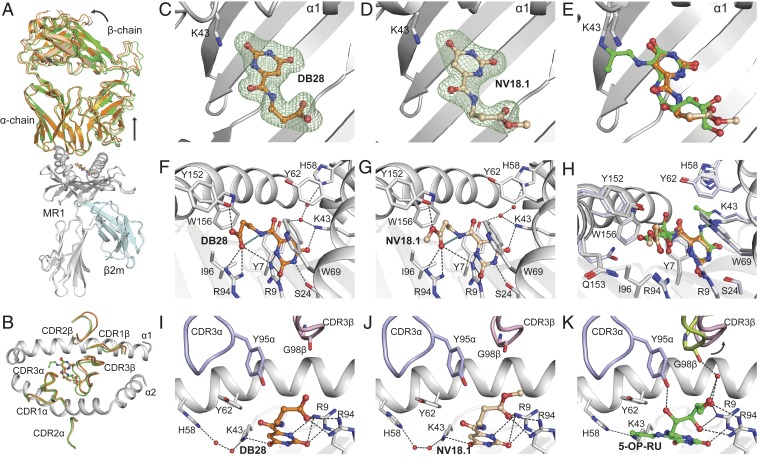
Ternary structures of AF-7-TCR-MR1 inhibitor complexes. (*A*) Overlay of the TCR-MR1 ternary structures of 5-OP-RU (PDB ID: 6PUC), DB28 and NV18.1 compounds. MR1 and β2m are colored gray and pale cyan, respectively. TCR chains and the ligands are colored as follows: 5-OP-RU, green; DB28, orange; and NV18.1, wheat. (*B*) The top view of MR1 cleft with the CDRα, CDRβ loops is shown as cartoon and colored as in *A*. (*C* and *D*) Omit maps contoured at 3σ (Fo − Fc map; green mesh) of DB28 (*C*) and NV18.1 (*D*) after stimulated annealing refinements in Phenix crystallography package. (*E*) Superposition of DB28, NV18.1, and 5-OP-RU metabolites within the binding cleft. (*F* and *G*) Detailed interaction of DB28 (*F*) and NV18.1 (*G*) within the A′-pocket of MR1 binding cleft. Intermolecular and intramolecular H-bonds are represented as black and deep-teal dashes, respectively. (*H*) Superposition of all pocket residues of DB28, NV18.1, and 5-OP-RU compounds highlighting small conformational differences of MR1 residues between various structures. The residues of MR1-DB28 and NV18.1 are colored gray, whereas the interacting residues of MR1-5-OP-RU are colored light blue. (*I*–*K*) Interaction between CDR3α loop and CDR3β loop with MR1 presenting DB28 (*I*) and NV18.1 (*J*) and 5-OP-RU (*K*), respectively. The CDR3α loop is colored blue in all panels, where the CDR3β loop is colored light pink in DB28 and NV18.1 structures, and lemon in 5-OP-RU complex.

To gain insight into the molecular basis underpinning MR1 down-regulation by DB28 and NV18.1, and despite the very low affinity of the interaction, as judged by SPR, we were able to crystallize the MAIT A-F7 TCR-MR1-DB28 and TCR-MR1-NV18.1 complexes, consistent with other structural reports with low-affinity ligands (26, 37). Both ternary structures were determined at 2-Å resolution and exhibited unambiguous electron densities within the MR1-antigen binding cleft and at the TCR-MR1-Ag interfaces (*SI Appendix*, Table S2 and Fig. 9). The overall topology of the two ternary complexes was very similar, whereby the MAIT TCR docks centrally above the MR1-antigen binding cleft, but with a little juxtapositioning of the TCR β-chain toward the F′ pocket compared with the TCR-MR1-5-OP-RU structure (PDB: 6PUC) (22, 37); this was accompanied by displacements and conformational changes within the Complementarity Determining Regions (CDR) loops of the TCR β-chain (CDRβ), while the CDR loop positions within the TCR α-chain were similar (Fig. 9 *A* and *B*).

As expected from their chemical compositions, neither DB28 nor NV18.1 forms a Schiff base with Lys43 of MR1; however, both ligands are clearly visible in the A′-pocket of MR1, as evidenced by unbiased omit maps of the ligands (Fig. 9 *C* and *D*), indicating strong sequestering of the ligands within the MR1 antigen-binding cleft. Here, the carbonyl group of the uracil ring of both ligands is H-bonded to Lys43, which results in Lys43 being positioned closer to the ring structure when compared with that of 5-OP-RU bound to MR1 (Fig. 9*E*). In addition, the uracil ring of DB28 and NV18.1 adopts a position reminiscent of the ring structure of 5-OP-RU (Fig. 9 *F*–*H*), whereby its positioning is largely governed by a network of contacts that include hydrophobic interactions with the MR1 aromatic cradle (Tyr7, Tyr62, Trp69, Ile96, and Trp156), as well as H-bonding to Lys43, Arg9, and Ser24 residues (Fig. 9 *F* and *G*). Interestingly, the orientation of the side-chain appendages of DB28 and NV18.1 within the A′-pocket differ from that of 5-OP-RU: both side-chains are constrained by an intramolecular H-bond between the amide NH of the side-chain and the carbonyl oxygen of the carboxylic acid (in DB28) or ester (in NV18.1; Fig. 9 *F* and *G*). This tilted conformation of the ligands’ side-chains is supported by hydrophobic interactions with Ile96, Trp69, and Trp156 residues, and H-bonding with the Tyr152 of the MR1 α2-helix. Further, the two side-chains lean toward and form H-bonds with Arg9 and Arg94 that protrude from the base of the MR1 A′-pocket. Changing the terminal carboxylic acid group of DB28 for an ester in NV18.1 causes no major structural changes within the MR1 pocket between both complexes. Collectively, a pattern of intermolecular hydrophobic and polar interactions is formed between DB28/NV18.1 ligands and the MR1 A′-pocket that sequester the ligands inside the cleft. No direct or water-mediated contacts were observed between the DB28/NV18.1 ligands and any of the TCR CDRα and CDRβ loops (Fig. 9 *I*–*K*), in agreement with the inability of these compounds to bind to the MAIT TCR (Fig. 8) and the capacity of DB28 to activate MAIT cells (Fig. 6).

## Discussion

During the past 7 y, several MAIT cell agonists and inhibitors have been identified (23, 24, 26). Stimulatory microbial ligands are characterized by the presence of a ribityl moiety, while their potency generally correlates with the formation of a Schiff base with Lys43 in the MR1 A′-pocket (38, 39). Formation of the Schiff base is thought to be the key molecular trigger for MR1 translocation to the cell surface, which is transiently observed upon ligand exposure (20). Through an in silico screen, we have identified additional MR1-binding ligands and now report the identity of a ligand that down-regulates MR1 cell surface expression. Unlike 5-OP-RU or Ac-6-FP, exposure to DB28 does not lead to MR1 translocation to the cell surface, and in primary monocytes, B cells, and both myeloid and B cell lines, it reduces MR1 basal levels of expression. Using epithelial cells expressing MR1 fused to GFP and intracellular staining for total MR1 proteins, we demonstrated that MR1 molecules are not degraded; rather, they are retained intracellularly in an EndoH-sensitive compartment, likely the ER/early Golgi. Consequently, DB28 is able to competitively inhibit MAIT cell activation by antigen-presenting cells pulsed with strong and weak synthetic agonists or infected with bacillus Calmette-Guérin or *E. coli*, bacteria that are both able to synthesize vitamin B2 metabolites that are strong MAIT cell agonists. Down-regulation of MR1 cell surface expression is observed with two monoclonal antibodies, recognizing different epitopes of correctly folded MR1 molecules, in agreement with the lack of complete maturation of MR1 molecules. The effect of DB28 is specific for MR1 molecules, as no down-regulation of MHC class I or CD1d molecules is observed. We also observed a trend of preferential inhibition of newly synthesized MR1 molecules, as demonstrated by lower MR1 down-regulation in the presence of the protein synthesis inhibitor cycloheximide or in cells expressing a doxycycline-inducible MR1 construct. This result suggests the existence of different compartments within the ER/early Golgi for distribution of MR1 ligands and might indicate differential association of MR1-loaded molecules with chaperones; for example, proteins of the peptide-loading complex. Thus, DB28-like molecules represent important tools for unraveling the molecular mechanisms of MR1-dependent antigen presentation. We investigated whether DB28 could be used in vivo to inhibit agonist-dependent MAIT cell activation. Despite the molar excess of DB28, we did not observe any inhibitory effect, likely because of the short half-life of the loaded complexes. Indeed, when murine bone marrow DC were used as antigen-presenting cells, DB28 could compete the activity of 5-OP-RU, thus ruling out a species-specific effect.

Structural studies confirmed the ability of DB28 to bind within the MR1 A′ pocket, with an overall topology reminiscent of 5-OP-RU. As predicted from its molecular structure, DB28 does not form a Schiff base with Lys43 in the MR1 A′ pocket. However, it is stabilized by a network of hydrophobic interactions and hydrogen bonds with the charged arginine residues. The lack of any inhibitory effect on Lys43Ala mutant MR1 molecules might be explained by the rapid egress of these molecules from the ER in the absence of any exogenous ligand (20). Other MR1 ligands (and weaker MAIT cell agonists) have been described that also lack the ability to form a Schiff base with Lys43; these include ribityl lumazines (22, 23) and diclofenac (26). While our results confirm and extend the observation that MR1 molecules are loaded in the ER (20) and the need for a Schiff base to trigger MR1 release to the cell surface, they also suggest that weaker, non-Schiff base-forming ligands may be loaded on the cell surface or in the recycling compartment. The ester analog of DB28, NV18.1, is less potent than DB28 in downregulating MR1 molecules. Consistently, the crystal structure of NV18.1-loaded MR1 molecules indicates that the extra methyl group in NV18.1 might impart more flexibility on the complex. Neither DB28 nor NV18.1 form direct contacts with the MAIT TCR, in agreement with their inability to activate MAIT cells in cellular assay, and consistent with the low-affinity binding of two MAIT TCRs to MR1 complexes bound to either DB28 or NV18.1.

Down-regulation of antigen-presenting molecules is a well-known strategy used by pathogens to evade MHC and CD1d-restricted T cell responses (40–42). Although to date we have not identified any microbial metabolite similar to DB28, we suggest that microbes may use evasion strategies targeting MR1 molecules, given the abundance of MAIT cells and their antimicrobial function (43). Similarly, it is tempting to speculate that self molecules similar to DB28 may physiologically regulate MR1 transit through the cell. Indeed, changes in cellular metabolites, for example, during neoplastic transformation, could potentially interfere with MR1 trafficking and modulate MAIT cell function in the tumor microenvironment.

In conclusion, we have identified a compound able to competitively down-regulate MR1 cell surface expression that may prove to be a useful tool compound for in vivo modulation of MAIT cell function. We have also identified additional MAIT cell agonists, which, similar to diclofenac (26), lack a ribityl moiety, and like lumazines (24), they are not predicted to form Schiff bases. Although weaker agonists than vitamin B2 intermediates, these compounds could be the starting point for structure-activity studies aimed at designing novel ligands that drive MAIT cell-dependent DC and B cell maturation (9, 44, 45).

## Materials and Methods

### Medium and Reagents.

The complete medium (CM) used throughout was RPMI 1640 (Gibco) for THP1 (ATCC), DMEM (Gibco) for BEAS2B, and IMDM (Gibco) for human MAIT cells. CM was supplemented with 2 mM l-glutamine, 1% nonessential amino acids, 1% sodium pyruvate, 1% pen/strep, 5 × 10^−5^ 2ME (all from Gibco), and serum: 10% FCS (Sigma) or 5% human AB Serum (Sigma) for MAIT cells. MAIT cell medium was supplemented with 1,000 U/mL recombinant human IL-2, produced in our laboratory, as previously described (9).

THP-1 cells (ATCC) and THP-1 overexpressing MR1 were maintained in CM. THP1 MR1 KO were previously described (36). THP1 cells were transduced with lentiviral particles generated in 293T cells transfected with lentiviral vectors encoding for full-length human MR1 (GenBank AJ249778.1), MR1 K43A (20), or GPI-linked MR1 (using the GPI sequence described in ref. 46).

BEAS2B WT cells were grown in DMEM supplemented with 10% heat-inactivated FBS (Gemini Bio-products) and 3.5 mM l-glutamine (Gibco). BEAS2B MR1 KO expressing doxycycline-inducible MR1 was previously described (34, 35).

Bacillus Calmette-Guérin (gift from Peter Sander) was grown in Middlebrook 7H9 broth supplemented with Middlebrook ADC (Thermo Fisher Scientific) and 0.2% glycerol. The bacteria were frozen down at 1.34 × 10^9^ CFU/mL, and thawed aliquots were passaged 10 times through a 27G syringe before infection. DH5α *E. coli* bacteria (Thermo Fisher) were grown overnight to stationary phase in Luria broth, extensively washed in PBS, and diluted to an OD600 of 0.5 (equivalent to about 400 million bacteria/mL). Acetyl-6-FP was purchased from Schircks laboratories and was resuspended in DMSO at 20 mg/mL. 5-A-RU was synthesized as described in the supplementary methods and was combined with 50 μM methylglyoxal (MG; Sigma) before each assay to obtain 5-OP-RU. MG was used alongside DMSO as a negative control in each stimulation assay. DB28 was purchased from Vitaslab.com (product STK870291) and resuspended in DMSO at 10 mg/mL. Cycloheximide and doxycycline were purchased from Sigma and resuspended in DMSO. All compounds were stored in small aliquots at −80 °C, protected from light.

### Generation of MAIT Cells and Antigen-Presenting Cells.

Blood was obtained from the UK National Blood Service. Human MAIT cells were isolated by cell sorting CD2 MACS enriched leukocytes with CD161 and Vα7.2 antibodies (Biolegend). In some experiments, antibodies to TRBV20.1 (Miltenyi) or TRBV13S2 (cone H132, Biolegend) were added to sort MAIT cell subsets. MAIT cells were grown for 3 wk in CM supplemented with IL-2. iNKT cells were generated and maintained as described (47).

The MR1-restricted CD8^+^ TRAV1-2^+^ T cell clone D426-G11 was previously described (48).

Jurkat expressing MAIT TCRs have been previously described (29).

THP-1 MR1-HA cells were generated transducing THP-1 cells with a lentivirus encoding for MR1-HA tagged molecules, cloned in the lentiviral vectors pHR-SIN with the following primers: forward: taa​ccg​AGA​TCT**cca​cc**atg​ggg​gaa​ctg​atg​gcg​ttc​c; reverse: (5′-3′) gct​aaG​CGG​CCG​Ctc​aAG​CGT​AAT​CTG​GAA​CAT​CGT​ATG​GGT​Atc​gat​ctg​gtg​ttg​gaa.

### Stimulation Assays.

THP-1 cells were plated at 50,000 cells/well in 96-well U-bottom plates in CM and incubated with MAIT cells (20,000 cells/well, in triplicate) in the presence or absence of different concentrations of DB19, 5-OP-RU, and DB28. DB28 (20 μg/mL) was added 2 h before 5-OP-RU unless otherwise stated. MAIT cell activation was assessed by IFN-γ ELISA (antibodies from Becton Dickinson) on supernatants harvested after 16 h. In some experiments, THP1 cells were incubated with 10 μM cycloheximide (Sigma) to inhibit protein synthesis, starting 30 min before addition of the ligands. In some experiments, THP1 cells were pulsed with the indicated concentrations of αGalCer (47) and incubated with iNKT cells at the same Effector:Target ratio described here.

BEAS2B WT cells were infected overnight with bacillus Calmette-Guérin at the indicated multiplicity of infection (MOI), harvested, washed, and 10,000 cells/well were plated in ELISPOT plates precoated with IFN-γ capture antibody (Mabtech, 1-DIK). Cells were treated with DB28 (20 μg/mL final concentration) or DMSO for 2 h before addition of phytohemagglutinin and 10,000 cells/well of the MAIT cell clone D426-G11, used from frozen. ELISPOTs were performed in RPMI (Gibco) supplemented with 3.5 mM l-glutamine (Gibco), 10% heat-inactivated human serum, and 50 μg/mL gentamycin (Gibco). IFN-γ spot-forming units were enumerated on an AID ELISPOT reader after development with an ALP antibody (Mabtech, 7-B6-1-ALP) after overnight incubation.

### MR1 Up-Regulation Assay.

THP1 cells overexpressing MR1 and CD1d were incubated for 5 to 7 h or overnight with 20 μg/mL of DB28/NV18.1, 5 μg/mL 5-OP-RU, or 1 μg/mL acetyl-6-FP. Cells were harvested and stained for cell surface MR1 (clone 26.5, Biolegend; clone 8F2F9, purified in house) or CD1d (clone 42.1, Biolegend). In some experiments, cells were washed and chased for the indicated amount of time before staining. Total MR1 contents were determined on fixed and permeabilized cells (Foxp3 kit; Thermo Fisher) with the rabbit MR1 polyclonal antibody (Proteintech, cat n 13260-1-AP), or with the anti-HA mouse monoclonal antibody (clone 2–2.2.14; Thermo Fisher) followed by PE-labeled anti-rabbit or anti-mouse antibodies (Thermo Fisher). Samples were acquired on a ×50 BD symphony machine and analyzed with Flowjo 10. Viability was assessed with live/dead staining (Aqua or near infrared), according to the manufacturer’s instructions (Thermo Fisher).

### Whole Blood Assay.

Freshly drawn blood was distributed in 5-mL polypropylene conical tubes (BD Falcon). One milliliter of blood was activated with 5-OP-RU (10 μg/mL) or *E. coli* at the indicated MOI in the presence or absence of DB28 (100 μg/mL). After overnight stimulation, cells were stained in Brilliant violet buffer (BD) with the following antibodies: BUV661 CD3 (UCHT1; BD), PE/Dazzle CD137 (VI C-7, Biolegend), antigen-presenting cell CD161 (HP-3G10, Biolegend), BV605 Vα7.2 (3C10, Biolegend), and BV510 CD19 (HIB19, Biolegend). Samples were acquired on a ×50 BD Symphony machine and analyzed with FlowJo 10. Viability was assessed with live/dead Aqua staining, according to the manufacturer’s instructions (Thermo Fisher). *E. coli* (DH5a; Thermo Fisher) was grown overnight in Luria broth medium and after extensive washing in PBS, OD_600_ was measured. An OD_600_ = 1 was considered equivalent to 5 × 10^8^ bacteria/mL

### BEAS2B Tet MR1-GFP Assays.

Cells were plated in 6-well plates at 70% confluence. MR1 expression was induced with 2 μg/mL doxycycline (Sigma) 16 to 24 h before addition of the ligands. Ligands were incubated either 7 h or overnight, in the presence or absence of doxycycline, before harvesting the cells for FACS analysis.

### Statistical Analysis.

Statistical analyses were performed with GraphPad Prism software, version 8. Comparisons were performed with *t* test, and differences with *P* < 0.05 were deemed significant.

### MR1 Docking.

Constraints imposed during docking included the presence of an aromatic ring at a distance suitable for aromatic interactions with Y7. Four hydrogen-bonding interactions were required out of the selected interactions formed by cocrystallized ligands in the complex structures used for the virtual screening. Poses lacking aromatic stacking interactions with Y7 residue of MR1 were excluded. Out of the top-scoring poses, the selection was based on favorable interactions with MR1/TCR residues and the presence of suboptimal contacts. In the case of compounds with acceptable poses, only the most favorable pose was included in the final selection.

### Protein Production and SPR Measurements.

Soluble A-F7 MAIT TCR (TRAV1-2-TRBV6-1), #6 (TRAV1-2-TRBV6-4) TCR, and human MR1-β2m-Ag were refolded from inclusion bodies and purified as described (22, 25). All SPR measurements were conducted in duplicate (*n* = 2) on a BIAcore 3000 instrument, as described previously (25, 37). For extended description, see *SI Appendix*, *Supplemental Experimental Procedures*.

### Crystallization, Structure Determination, and Refinement.

A-F7 TCR was mixed with MR1-β2m-Ag in 1:1 ratio, and ternary complex crystals were obtained by hanging drop crystallization, as established previously (22). Data were collected at the Australian Synchrotron Facility, processed and refined with standard software packages. For an extended description, see *SI Appendix*, *Supplemental Experimental Procedures*.

### Accession Numbers.

The coordinates of the ternary complexes of MAIT A-F7 TCR-MR1-DB28 and TCR-MR1-NV18.1 have been deposited in the Protein Data Bank under accession codes 6PVC and 6PVD.

## Supplementary Material

Supplementary File
