# Supplementary material for: Ligand-dependent downregulation of MR1 cell surface expression
Source: Proc Natl Acad Sci U S A. 2020 Apr 27;117(19):10465–75. doi: 10.1073/pnas.2003136117 (PMC7229755; doi:10.1073/pnas.2003136117)
Supplement: Supplementary File [file pnas.2003136117.sapp.pdf]

Supplementary Information for

Ligand-dependent downregulation of MR1 cell surface expression

Mariolina Salio[1#], Wael Awad[2#], Natacha Veerapen[3#], Claudia Gonzalez-Lopez[1], Corinna Kulicke[4,5], Dominic Waithe[6], Anne W.J. Martens[1], David M. Lewinsohn[4,5], Judith V. Hobrath[7], Liam R. Cox[8], Jamie Rossjohn[2,9,10*], Gurdyal S. Besra[3*] and Vincenzo Cerundolo[1*†]

[1]Medical Research Council Human Immunology Unit, Medical Research Council Weatherall Institute of Molecular Medicine, University of Oxford, OX3 9DS, UK

[2]Infection and Immunity Program and Department of Biochemistry and Molecular Biology, Biomedicine Discovery Institute, Monash University, Clayton, VIC, 3800, Australia

[3]Institute of Microbiology and Infection, School of Biosciences, University of Birmingham, Edgbaston, Birmingham, B15 2TT, UK

[4]Division of Pulmonary and Critical Care Medicine, Department of Medicine, Oregon Health & Science University, Portland, 97239, Oregon, USA

[5]Portland Veterans Administration Healthcare System, Research Department, Portland, 97239, Oregon, USA

[6] Medical Research Council Centre for Computational Biology, The Wolfson Imaging Centre at the Medical Research Council Weatherall Institute of Molecular Medicine, University of Oxford, OX3 9DS, UK

[7]Drug Discovery Unit, College of Life Sciences, University of Dundee, Dundee, DD1 5EH, UK

[8]School of Chemistry, University of Birmingham, Edgbaston, Birmingham, B15 2TT, UK

[9]Australian Research Council Centre of Excellence in Advanced Molecular Imaging, Monash University, Clayton, Victoria 3800, Australia.

[10] Institute of Infection and Immunity, Cardiff University School of Medicine, Heath Park, Cardiff, CF14 4XN, UK.

[#]Joint first authors

[*]Joint senior authors

[†]Deceased 7 January 2020

Correspondence: mariolina.salio@imm.ox.ac.uk, g.besra@bham.ac.uk

This PDF file includes:

Supplementary material and methods
Figures S1 to S11
Tables S1 to S2

www.pnas.org/cgi/doi/10.1073/pnas.2003136117

Computational Method.

For *in silico* screening we utilized two crystal structures of the mucosal-associated invariant T-cell antigen receptor (MAIT TCR) in complex with MR1-restricted activator antigens: the human structure at 1.9 Å resolution (PDB code 4L4V) containing 7-hydroxy-6-methyl-8-D-ribityllumazine (RL-6-Me-7-OH) [1], and the structure of human MAIT TCR complexed with a humanized bovine MR1 presenting a highly potent activator antigen, reduced 6-hydroxy-methyl-8-D-ribityllumazine (rRL-6-CH_2OH), determined at 3.26 Å resolution (PDB code 4LCC) [2]. We selected these crystal structures for docking because the co-crystallized ligands show flipped or opposite orientations of their ring structures in the MR1 binding pocket, leading to slightly different conformations of the binding site induced by their binding. Both antigens form an extensive hydrogen-bonding interaction network in the complex structure. Common to both ligands are hydrogen-bonding interactions with S24, K43, R94, Y152 in MR1 and Y95 in CDR3α, while distinct interactions involve, for example, the CDR3β residues E99 (in 4LCC) and MR1 residues R9, Q153 (in 4L4V). Another important contributor to their binding are π-stacking interactions with the MR1 residue Y7. Compounds for docking were selected from the following commercial libraries: Asinex, Chembridge, Enamine, InterBioScreen, Life Chemicals, Maybridge, Otava, SPECS, TimTec and Vitas (updated in May, 2015). Two filtering methods implemented in the Schrödinger software package were applied: the REOS (Rapid Elimination of Swill) and PAINS (Pan Assay Interference) filters, resulting in 4,858,131 compounds from all commercial libraries after filtering. A subset of 44,022 compounds were further selected for docking runs based on searches for fragment size sub-structures s1-s20. Commercial compounds were docked into both MAIT TCR-MR1 crystal structures (PDB codes 4L4V and 4LCC). Constraints imposed during docking included the presence of an aromatic ring at a distance suitable for aromatic π-stacking interactions with Y7. Four hydrogen-bonding interactions were required out of the selected interactions formed by co-crystallized ligands in the two complex structures used for virtual screening. Results of the two docking runs were analyzed independently. Poses lacking aromatic π-stacking interactions with the Y7 residue of MR1 were excluded. Out of the top-scoring poses, the selection was based on favourable interactions with MR1/TCR residues, and the presence of suboptimal contacts. In the case of compounds with acceptable poses at both crystal structures, only the most favourable pose was included in the final selection.

Docking method applied for virtual screening.

The MAIT TCR-MR1 crystal structures (PDB codes 4L4V and 4LCC) were prepared using the Protein preparation wizard implemented in the Schrödinger package. Following addition of hydrogens, structures including co-crystallized ligands were relaxed through restrained minimization, applying default parameters to a maximum of 0.3 root-mean-square deviation between the refined and input crystal structures. Commercial compounds selected for docking were prepared using LigPrep in Schrödinger at pH = 7.4. For all docking runs, the Glide docking method as implemented in Schrödinger (version 2015-2) was used. Default parameters were applied, except for increasing the number of poses kept for the initial phase of docking to 7500, and poses selected for energy minimization to 600. Constraints applied are as follows: based on hydrogen-bonding interactions present in crystal structures (PDB codes 4L4V and 4LCC), ten possible hydrogen-bonding constraints were defined involving MR1 residues R9, S24, R94, Y152, Q153 and the MAIT TCRα residue Y95. Out of these, four hydrogen-bonding interactions were required, except for docking sets s4 – s8 where three interactions were required. The presence of an aromatic ring within 1.5 Å from the 6-membered ring closest to the MR1 residue Y7 in the co-crystallized ligand was required during docking. Compounds were docked into both crystal structures. In the case of compounds that were selected based on docking at both crystal

structures, only one pose per compound was selected, showing most favourable interactions with MAIT TCR-MR1 residues considering either crystal structures.

Fragment size structural motifs for the selection of commercial subsets for docking.

4,858,131 commercial compounds were searched for structures containing the motifs s1 – s20 (Figure S1). Substructures s1 – s11 were designed based on structural elements of ligands co-crystallized in MR1 – MAIT TCR complexes that contribute favourable interactions with MR1/TCR residues. A small diversity set of six common structural motifs s12 – s17 was also selected. These motifs contain hydrogen-bond acceptor/donor groups in 3D spatial arrangements that correspond to the arrangement of hydroxyl groups of co-crystallized riboflavin metabolites near CDR3 loops. As listed in Table S1, some of the obtained sets were further filtered to require the presence of at least 2 or 3 hydrogen-bond donor/acceptors, considering that co-crystallized ligands participate in a network of hydrogen-bonding interactions with MR1/TCR residues. In order to increase structural diversity, pyridine and simple pyrazines were also defined as substructures (s18 – s20), coupled with the requirement for higher number of hydrogen-bond donors/acceptors as listed in Table S1. Only heavy atoms were considered in all substructure searches and duplicate compounds between subsets were removed. Substructure searches based on s1 – s20 resulted in a total of 44,022 unique compounds which were further utilized for docking simulations. Physicochemical properties of commercial compounds were computed using QikProp (Schrödinger software) only to flag scaffolds with potentially less desirable properties.

Reagents and characterization.

All reagents and solvents were purchased from Sigma-Aldrich and used without further purification unless otherwise indicated.

Synthesis of 5-A-RU.

Several syntheses of 5-A-RU have been reported in the literature [3, 4, 5, 6, 7, 8, 9], but the compound still remains challenging to prepare and handle because of its instability. The sensitivity of 5-A-RU to photodegradation and oxidation to generate multiple products is well documented [7, 10]. Accordingly, 5-A-RU is rarely isolated and stored for further use [9, 10, 11]. It is usually freshly prepared by the reduction of 5-nitro-6-(ribitylamino) pyrimidine-2, 4-(1*H*, 3*H*)-dione, or the 5-nitroso analogue, before immediate use in chemical reactions or biological and immunological assays [9, 11]. Immunological-based assays are sensitive to contaminants such as residual reducing agents; hence it is desirable to use compounds as pure as possible. The fully protected nitro derivative **1** was prepared following Cushman's [3] synthetic route (Figure S2A). Treatment with TBAF, followed by concomitant palladium-catalyzed hydrogenation of the nitro group and hydrogenolysis of the benzyl ethers in **2** proceeded smoothly, to afford the product 5-A-RU. However, despite our best efforts to exclude light and air, upon filtration and evaporation of the solvent, the isolated amine gradually turned pink. While the NMR spectroscopic and mass spectrometric data confirmed that the pink solid consisted of 5-A-RU, the nature of the pink colour could not be determined unambiguously. Nonetheless, we did establish that the pink colour caused the compound to fluoresce and while its modulatory activity towards MAIT cells was comparable to that of a non-fluorescent batch of 5-A-RU (Figure S2B), the pink 5-A-RU was not be used in other immunological-based experiments, such as the preparation of MR1-loaded tetramers. To minimize manipulations and circumvent the formation of the pink impurity, we resolved to reducing the nitro group in compound **1** to the amine **3** before global deprotection. We reasoned that the uracil ring in the protected derivative **3**, being trapped as the aromatic imidic form, would be unable to tautomerize to the less stable amide form. Amine **3** proved to be stable enough to be purified by column

chromatography. Treatment of amine **3** with degassed 1 M HCl in ethanol under argon, followed by evaporation of the solvent and the volatile side-products under reduced pressure in the dark, afforded the hydrochloride salt of 5-A-RU as an off-white solid, which could be stored at 4 °C for a prolonged period. The spectroscopic data of the latter were consistent with those reported in the literature [3, 9, 11]. Our synthesis highlights that benzyl ethers on the uracil ring are reasonably labile and cleavable by acid. This alternative synthetic pathway is also to our knowledge the first report of the successful isolation of 5-A-RU. The relative potency of four different batches of 5-A-RU/MG, prepared by both methods, is shown in Figure 2. Comparable secretion of IFN-γ by MAIT cells was elicited by all four batches, even after prolonged storage at –80 °C (Figure S2B).

Immunoprecipitation.

Cells were washed twice in cold PBS and lysed for 30 min on ice (100 µL of lysis buffer per 10 million cells) in lysis buffer (20 mM Tris-HCl pH 7.5, 150 mM NaCl, 1 mM EDTA pH 8.0, 1% Triton X-100, 1 mM EGTA, 2.5 mM sodium pyrophosphate) containing protease inhibitors. Cytoplasmic lysates were obtained after centrifugation of the whole-cell lysates for 10 min at 13000 rpm at 4 °C, and then they were precleared with Protein G agarose beads (Pierce) overnight at 4 °C in a rotatory mixer. Incubation of the monoclonal antibody anti-MR1 (26.5) or isotype control antibody (mouse monoclonal anti-TNP IgG2a antibody, ATCC) with the Protein G agarose beads was carried out overnight at 4 °C at a final concentration of 40 µg/ml. The precleared lysates were incubated with the antibodies bound to protein G agarose beads for 5 h at 4 °C in a rotatory mixer, the beads were washed 3 times in cold lysis buffer and proteins were eluted from beads by adding 2X sample loading buffer and heating at 95 °C for 5 min. If samples were treated with EndoH, after the third wash of the beads with lysis buffer, the beads were incubated with Glycoprotein Denaturing Buffer (NEB) and incubated at 100 °C for 10 min. After centrifugation, the supernatant was collected and incubated with Glycobuffer 3 (NEB) and EndoHf (NEB) for 1.5 h at 37 °C. Sample loading buffer was added to the reaction mixture and the sample was heated at 95 °C for 5 min before loading it in 4-12% acrylamide gel. The gel was transferred to a PVDF membrane (Bio Rad, Trans-Blot Turbo Transfer Pack) using the Bio Rad Trans-Blot Turbo system following the manufacturer's instructions. After the transfer, membranes were washed in PBS and blocked with PBST (PBS + 0.1% Tween 20) + 5% semi skimmed milk for 1 h at RT. After blocking, the membranes were incubated with MR1 polyclonal antibody (Proteintech, cat n 13260-1-AP), mouse monoclonal antibody anti-β actin (Santa Cruz Biotechnology, sc-47778) in blocking solution overnight at 4 °C, washed 4 times in PBST and incubated with goat anti-rabbit IRDye 800CW antibody (Li-Cor, cat n 925-32211) and goat anti-mouse IRDye 680RD antibody (Li-Cor, cat n 925-68070) in blocking solution for 1 h at 20 °C. Membranes were washed 4 times in PBST and once with PBS. Membranes were dried between 3MM Whatman paper and then scanned using the Odyssey® Near-Infrared imaging system. To quantify the proteins, rectangle shapes were drawn around the bands and the signal intensity was calculated subtracting the signal of the background from the total intensity of each band.

Confocal microscopy.

Cells were washed twice in DPBS (PBS containing Mg^{2+} and Ca^{2+}) (Lonza) and seeded on clean sterile 13 mm diameter covers that were pre-coated with poly-D-lysine hydrobromide (Sigma) for 1 h at 20 °C. Cells were incubated overnight at 37 °C 5% CO_2. The next day, cells were washed twice in DPBS and fixed in freshly prepared 4% PFA (Thermo Scientific) in PBS for 20 min at RT. After washing the cells twice with DPBS, cells were permeabilized with 0.1% Triton X-100 in DPBS for 20 min at RT (20 °C), washed again twice with DPBS and incubated with blocking solution (2% BSA, 10% FCS, 5% HS, human immunoglobulins, DPBS) for 30 min at RT (20 °C). Covers were transferred to a humid chamber and incubated with a polyclonal antibody anti-calnexin antibody (Millipore, cat AB2301) and a mouse monoclonal anti-MR1 antibody (26.5) or a polyclonal rabbit anti-MR1 antibody (Proteintech, cat n. 13260-1-AP) overnight at 4 °C in darkness. After washing

the covers 5 times with DPBS and once with sterile water, covers were mounted on clean microscopy slides with Vectashield containing DAPI. Slides were stored at 4 °C. Images were acquired using a Zeiss 780 inverted confocal microscope equipped with LSM software.

Colocalization analysis.

To analyze the colocalization of two proteins, each confocal image was opened in ImageJ (version 2.0.0 -rc- 65/1.52i) and the cells of interest were drawn around using the polygon tool. The regions of interest (ROI) were added to the ROI Manager before running a custom-written macro (available upon request) which measures the colocalization between two channels in a region. The algorithm works through each of the selected regions and give us the Pearson's values for each cell. These values were copied to an Excel spreadsheet and then plotted in Prism 7 for MAC OS X to produce the graphs and apply statistical analysis.

Protein Expression, Refold and Purification of MR1-β2m-Ag and MAIT TCRs.

Human MR1-β2m was refolded in the presence of ligand as described previously[12]. Soluble A-F7 (TRAV1-2-TRBV6-1) and TRAV1-2-TRBV6-4 (#6) MAIT TCRs[13] were refolded from inclusion bodies as described previously [1, 14]. Refolded MR1-Ag and TCR proteins were purified by sequential crude anion exchange, size exclusion chromatography and finally HiTrap-Q HP anion exchange. The purity of the resulting protein was assessed using SDS-PAGE. DB28 and NV18.1 compounds didn't refold MR1 to sufficient amount *in vitro* (data not shown), therefore a ligand exchange approach was used to load MR1 with those ligands as described previously[15]. Briefly, the ligand was added to MR1-Ag solution at concentration of 0.5-3 mM, incubated overnight at 4 °C, then aggregated proteins were removed by size exclusion chromatography. This approach was optimized to produce MR1 loaded with 5-OP-RU, Ac-6-FP, DB28 and NV18.1 ligands for all experiments.

Surface Plasmon Resonance (SPR) Measurements.

All SPR measurements were conducted in duplicate (n=3) on a BIAcore 3000 instrument using HBS buffer (10 mM HEPES-HCl pH 7.4, 150 mM NaCl, and 0.005% surfactant P20) as described previously[14]. Biotinylated C-terminal Cys-tagged-MR1-Ag was immobilized on SA-Chips with a surface density of ~3000 response units (RU). The flow cell of the SA chip (FC1) was loaded with MR1-weak ligand, and the binding value was subtracted as indicative of non-specific binding. FC2 was loaded with MR1-5-OP-RU as a positive control and FC3 and FC4 were loaded with two different MR1-Ags. Various concentrations (0-400 μM) of two different MAIT TCRs: A-F7 (TRAV1-2-TRBV6-1) and TRAV1-2-TRBV6-4 were injected over the chip at 5 μL/min at 25 °C and equilibrium data were collected. The SPR sensograms, equilibrium curves and steady state K_D values (μM) were prepared in Prism 7.

Protein Crystallization, Structure Determination and Refinement.

Purified A-F7 TCR was mixed with MR1-β2m-Ag in a 1:1 molar ratio at a concentration of 4-5 mg/mL and kept on ice for 2 h. Hanging drop crystallization was employed to produce crystals of the complexes with a precipitant consisting of 100 mM Bis –Tris Propane (BTP; pH 6.1 - 6.7), 10-20% PEG3350 and 200 mM sodium acetate as established previously[1]. Complex crystals formed over 2-7 days at 20 °C and were flashed frozen in liquid nitrogen after quick soaking in reservoir solution with 10-15% glycerol for cryo-protection. X-Ray diffraction data were collected at 100 K on the Australian Synchrotron at either MX1 or MX2 beamlines. Diffraction images were processed using XDS[16] and programs from the CCP4 suite[17] and Phenix package[18]. Both ternary complex structures were determined by Molecular Replacement using PHASER[19], where modified TCR-

MR1 ternary complex (PDB; 4L4T) was used as the search model. Model building was performed in COOT [20], accompanied with iterative rounds of refinement using Phenix.refine[18]. The Grade Webserver and Phenix tools were used to build and to generate ligand restraints. The models were validated using MolProbity[21] and graphical representations were generated using PyMOL Molecular Graphics System, Version 1.8, (Schrödinger, LLC, New York, NY).

***In vivo* and *in vitro* activity of DB28 in mice.** Animal experiments were performed under an appropriate United Kingdom Home Office licence, with ethical approval from the University of Oxford. C57BL/6 mice (purchased from Jackson laboratories) were injected in the lateral vein with 45, 15 or 5 nmol of 5-OP-RU with or without 440 nmol DB28 in 100 μL of PBS. After 24 hours, the mice were euthanized and MAIT cell frequency was determined by MR1 tetramer staining in a single cell suspension of splenocytes, as described[22]. The MR1 tetramer technology was developed jointly by Dr. James McCluskey, Dr. Jamie Rossjohn, and Dr. David Fairlie, and the material was produced by the NIH Tetramer Core Facility as permitted to be distributed by the University of Melbourne.

BMDC from C57BL/6 WT or MR1 KO mice were prepared as previously described[23] pulsed with 5-OP-RU (150 ng/mL) with or without DB28 (20 μg/mL) and incubated with human MAIT cells.

Table S1. Number of compounds in sets obtained from substructure searches based on s1 – s20 (No. in set), and additional filtering criteria applied requiring hydrogen-bond (H bond) acceptor/donor groups. NA: No additional filtering criteria used for the substructure search.

Substructure	No. in set	No. of H bond acceptors	No. of H bond donors
**s1**	55	NA	NA
**s2**	1134	≥ 3	≥ 2
**s3**	312	≥ 3	≥ 2
**s4**	4361	NA	NA
**s5**	2698	NA	NA
**s6**	3308	NA	NA
**s7**	5301	NA	NA
**s8**	7286	NA	NA
**s9**	6758	NA	NA
**s10**	2153	NA	NA
**s11**	155	NA	NA
**s12**	35	NA	≥ 3
**s13**	386	NA	≥ 3
**s14**	1069	NA	≥ 3
**s15**	447	NA	≥ 3
**s16**	116	NA	≥ 3
**s17**	1055	NA	≥ 3
**s18**	2529	≥ 6	≥ 3
**s19**	4492	≥ 6	≥ 3
**s20**	372	≥ 6	≥ 3

Table S2. Diffraction data collection and refinement statistics.

	**A-F7 TCR-MR1-DB28**	**A-F7 TCR-MR1-NV18.1**
**Resolution range (Å)**	45.42 - 1.96 (2.03 - 1.96)	53.49 - 2.14 (2.216 - 2.14)
**Space group**	C 1 2 1	C 1 2 1
**Unit cell**<br>**a, b, c (Å)**<br>**α, β, γ (°)**	214.291 69.834 141.618<br>90 103.319 90	216.057 70.112 142.874<br>90 103.97 90
**Total reflections**	622310 (62376)	553535 (57117)
**Unique reflections**	146278 (14549)	113315 (11169)
**Multiplicity**	4.3 (4.3)	4.9 (5.1)
**Completeness (%)**	99.83 (99.86)	98.70 (98.17)
**Mean I/sigma(I)**	15.89 (1.79)	13.15 (2.28)
**Wilson B-factor**	35.03	42.56
**R-merge**	0.05599 (0.8349)	0.06992 (0.7561)
**R-pim**	0.03073 (0.4573)	0.0351 (0.3684)
**CC1/2**	0.999 (0.736)	0.998 (0.799)
**R-work**	0.1694 (0.2697)	0.1687 (0.2299)
**R-free**	0.2110 (0.3128)	0.1985 (0.2467)
**Non-hydrogen atoms**	14903	14252
**macromolecules**	13234	13033
**ligands**	49	51
**solvent**	1620	1168
**Protein residues**	3230	2487
**RMS(bonds) (Å)**	0.009	0.003
**RMS(angles) (°)**	1.01	0.62
**Ramachandran favored (%)**	97.79	99.11
**Ramachandran allowed (%)**	2.21	0.76
**Ramachandran outliers (%)**	0.00	0.13
**Average B-factor**	46.26	49.42
**macromolecules**	45.50	49.17
**ligands**	39.12	46.79
**solvent**	52.68	52.28
	**A-F7 TCR-MR1-DB28**	**A-F7 TCR-MR1-NV18.1**
**Resolution range (Å)**	45.42 - 1.96 (2.03 - 1.96)	53.49 - 2.14 (2.216 - 2.14)
**Space group**	C 1 2 1	C 1 2 1

**Unit cell**<br>**a, b, c (Å)**<br>**α, β, γ (°)**	214.291 69.834 141.618<br>90 103.319 90	216.057 70.112 142.874<br>90 103.97 90
**Total reflections**	622310 (62376)	553535 (57117)
**Unique reflections**	146278 (14549)	113315 (11169)
**Multiplicity**	4.3 (4.3)	4.9 (5.1)
**Completeness (%)**	99.83 (99.86)	98.70 (98.17)
**Mean I/sigma(I)**	15.89 (1.79)	13.15 (2.28)
**Wilson B-factor**	35.03	42.56
**R-merge**	0.05599 (0.8349)	0.06992 (0.7561)
**R-pim**	0.03073 (0.4573)	0.0351 (0.3684)
**CC1/2**	0.999 (0.736)	0.998 (0.799)
**R-work**	0.1694 (0.2697)	0.1687 (0.2299)
**R-free**	0.2110 (0.3128)	0.1985 (0.2467)
**Non-hydrogen atoms**	14903	14252
**macromolecules**	13234	13033
**ligands**	49	51
**solvent**	1620	1168
**Protein residues**	3230	2487
**RMS(bonds) (Å)**	0.009	0.003
**RMS(angles) (°)**	1.01	0.62
**Ramachandran favored (%)**	97.79	99.11
**Ramachandran allowed (%)**	2.21	0.76
**Ramachandran outliers (%)**	0.00	0.13
**Average B-factor**	46.26	49.42
**macromolecules**	45.50	49.17
**ligands**	39.12	46.79
**solvent**	52.68	52.28

Statistics for the highest-resolution shell are shown in parentheses.

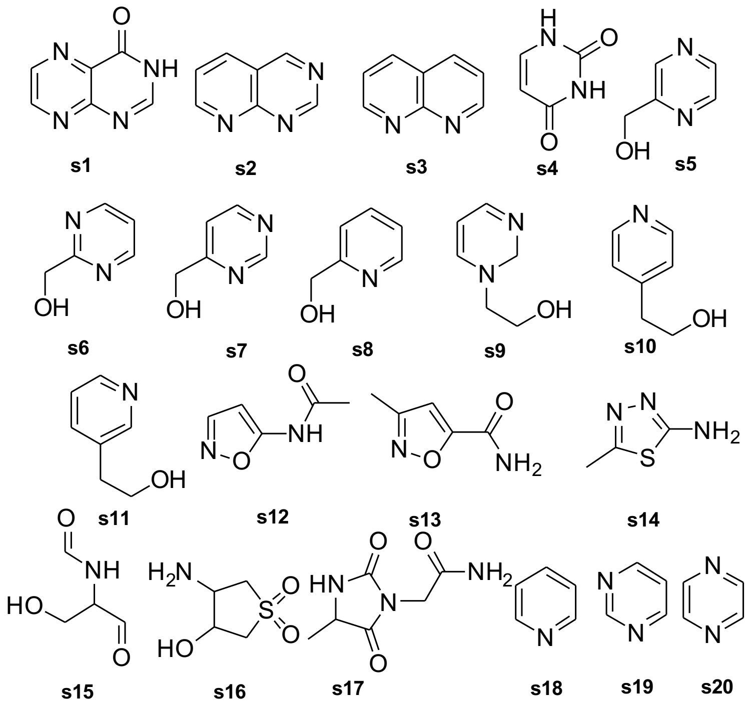

Figure S1. Fragment size scaffolds used for substructure-based pre-filtering of commercial libraries.

A

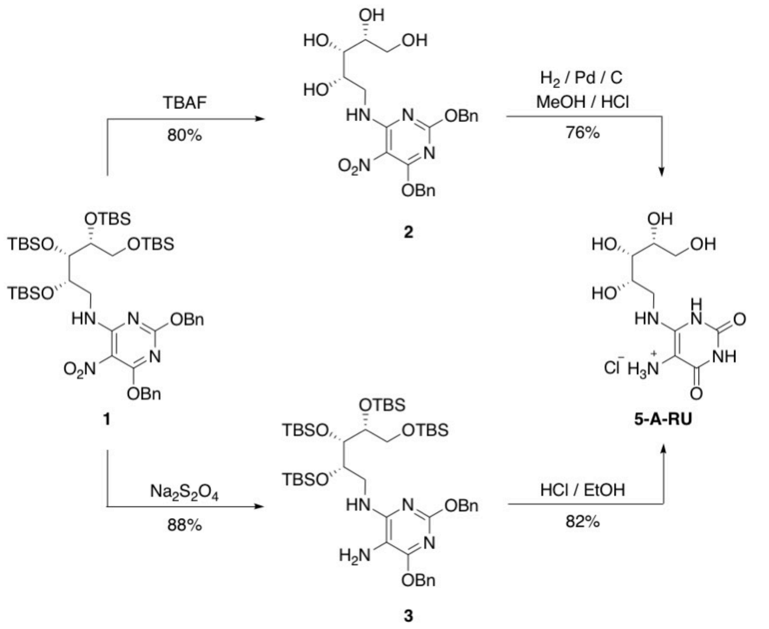

B

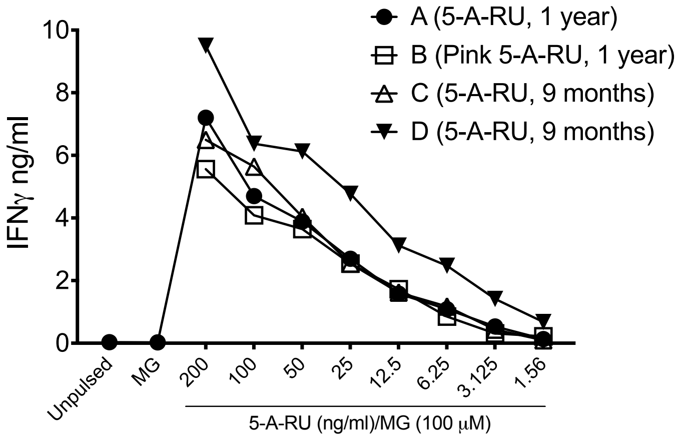

Figure S2. Synthesis and validation of 5-A-RU.

A) Synthesis of 5-A-RU. B) Validation of 5-A-RU activity. THP1 cells were pulsed with four different batches of 5-A-RU at the indicated concentrations in the presence of constant methylglyoxal (MG) (100 µM) to obtain 5-OP-RU and were incubated with MAIT cells for 36 hours. IFN-γ was measured in the supernatant after 36 hours of co-culture.

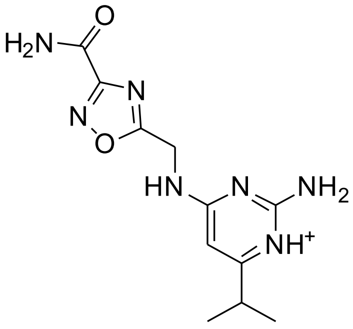

DB-5

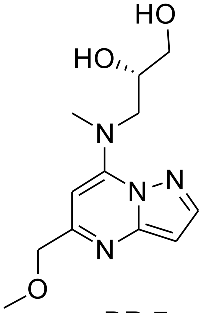

DB-7

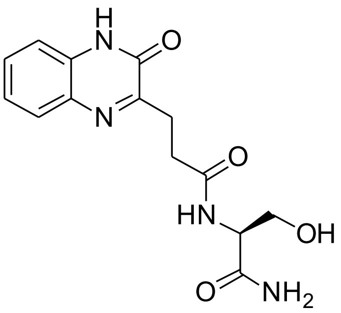

DB-19

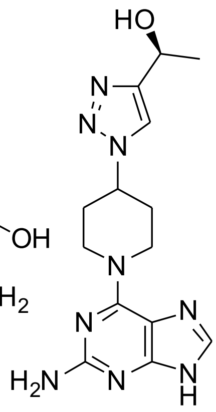

DB-15

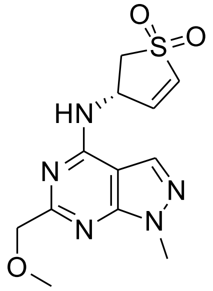

DB-8

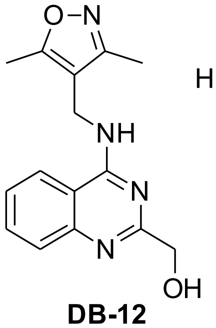

DB-12

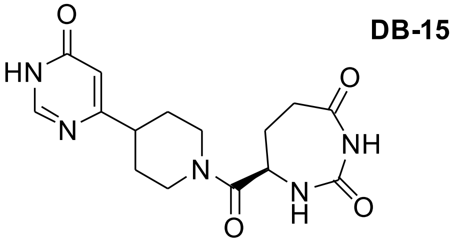

DB-23

DB-5: 2-amino-4-(((3-carbamoyl-1,2,4-oxadiazol-5-l)methyl)amino)-6-isopropylpyrimidin-1-ium

DB-19: (*S*)-*N*-(1-amino-3-hydroxy-1-oxopropan-2-yl)-3-(3-oxo-3,4-dihydroquinoxalin-2-yl)propanamide

DB-15:(*S*)-1-(1-(1-(2-amino-9*H*-purin-6-yl)piperidin-4-yl)-1*H*-1,2,3-triazol-4-yl)ethan-1-ol

DB-7:(*S*)-3-((5-(methoxymethyl)pyrazolo[1,5- α]pyrimidin-7-yl)(methyl)amino)propane-1,2-diol

DB-8:(*S*)-3-((6-(methoxymethyl)-1-methyl-1 *H*-pyrazolo[3,4-*d*]pyrimidin-4-yl)amino)-2,3-dihydrothiophene 1,1-dioxide

DB-12: (4-(((3,5-dimethylisoxazol-4-yl)methyl)amino)quinazolin-2-yl)methanol

DB-23:(*R*)-7-(4-(6-oxo-1,6-dihydropyrimidin-4-yl)piperidine-1-carbonyl)-1,3-diazepane-2,4-dione

Figure S3. Structural formulae and names of the DB series of MAIT cell agonists.

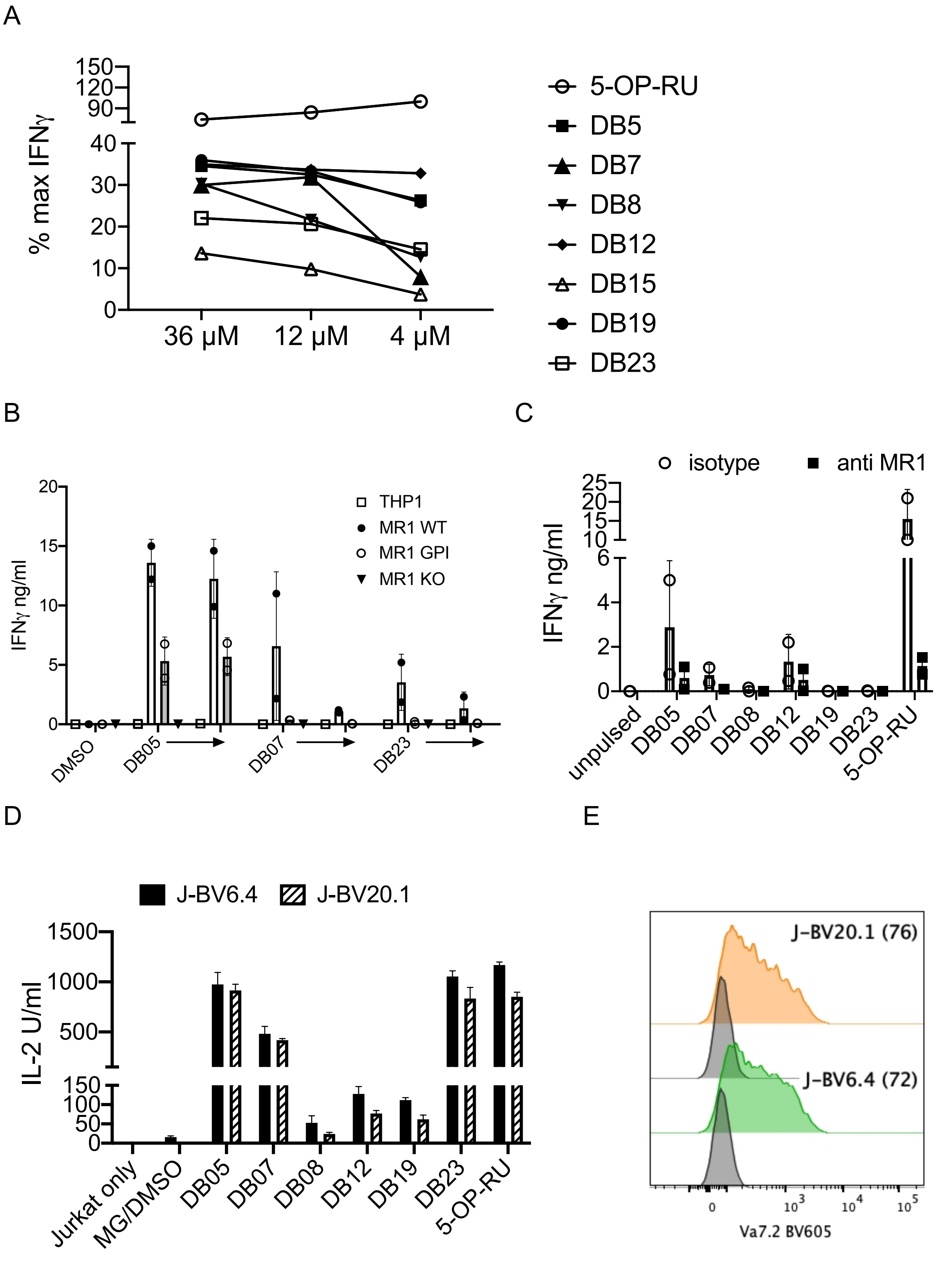

Figure S4. MAIT cell stimulation by the DB series of agonists. A) MAIT cell stimulation by seven DB agonists. THP1-MR1 cells were pulsed with the indicated concentrations of DB05, DB07, DB08, DB09, DB12, DB15, DB19 or DB23, and incubated with MAIT cells for 36 hours. IFN-γ was

measured in the supernatant after 36 hours of co-culture. Plotted is the % IFNγ secretion, normalized to 4 μM 5-OP-RU. One experiment representative of two. B) Un-transduced THP1, THP1 overexpressing MR1 WT or GPI linked and MR1-KO THP1 were pulsed with 20 or 10 μg/mL of the indicated compounds (right pointing arrow on the graph) and incubated with MAIT cells from two different donors. IFN-γ was measured in the supernatants after 36 hours of co-culture. Each dot represents the average of duplicate wells from one donor. C) Monocyte-derived dendritic cells were pulsed with 20 μg/mL of the indicated compounds and incubated with MAIT cells from two different donors, in the presence of 20 μg/mL isotype control or anti MR1 26.5 blocking antibodies. IFN-γ was measured in the supernatants after 36 hours of co-culture. Each dot represents the average of duplicate wells from one donor. D) THP1-MR1 were pulsed with the indicated compounds (40 μg/mL DB compounds; 200 ng/mL 5-A-RU and 100 μM methylglyoxal (MG)) and incubated with Jurkat JRT3 transduced with the MAIT TCR expressing the indicated TCRβ chains. IL-2 was measured in the supernatant after 36 hours of co-culture. Data are mean +/- SD of triplicate wells. E) TCR Vα7.2 surface expression in the two Jurkat lines tested (Geo MFI in brackets). D and E, one experiment representative of three.

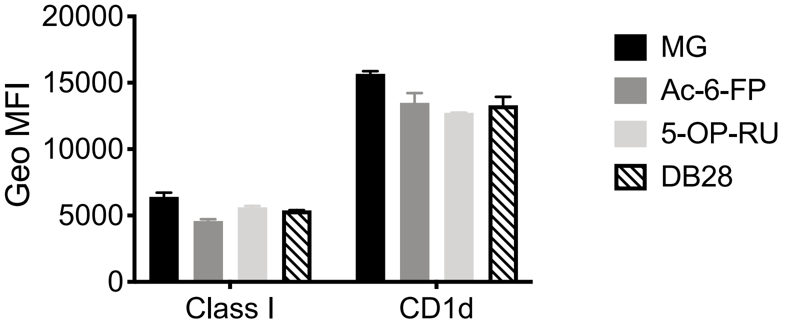

Figure S5. DB28 does not affect MHC Class I or CD1d cell surface expression.

THP1 cells were incubated overnight with MG, Ac-6-FP, 5-OP-RU or DB28 (as described in Figure 2B), and stained for cell-surface MHC Class I or CD1d. Histogram bars depict average expression of cell surface MHC Class I and CD1d (average Geo MFI of duplicate wells +/– SD), after gating on live, single cells. One experiment representative of three.

A

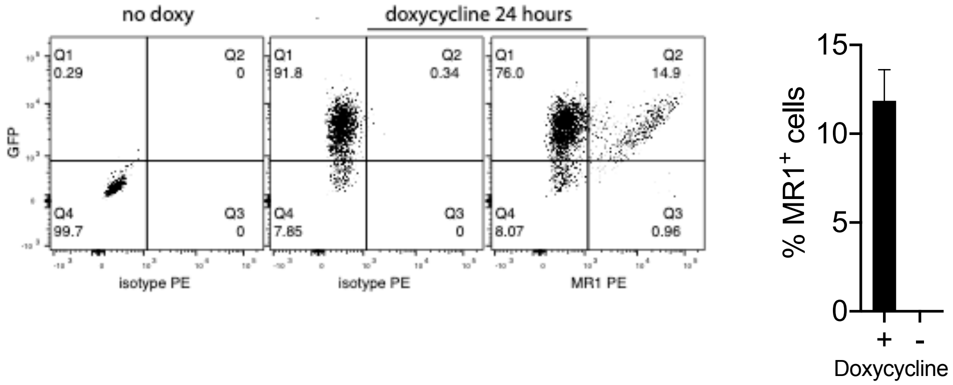

B

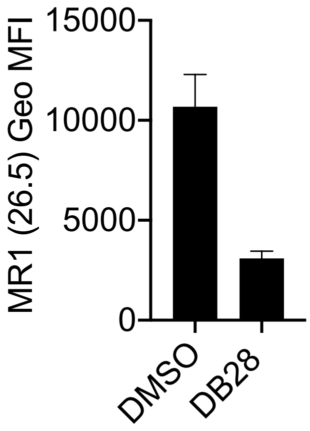

C

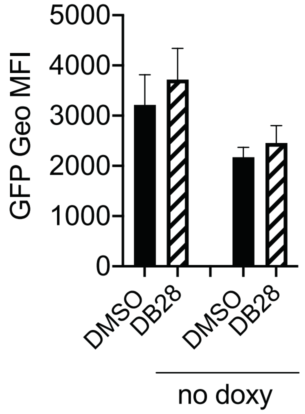

Figure S6. Inducible MR1 expression in BEAS2B cells. A) BEAS2B tet MR1-GFP cells were treated with 2 µg/mL doxycycline for 24 hours, and GFP and surface MR1 expression were detected by flow cytometry. Representative FACS plots (left) and cumulative surface MR1 expression (right) in 5 experiments, mean +/– SD. B) BEAS2B expressing MR1-GFP tagged were pulsed overnight with DB28 (20 µg/mL) before staining with anti MR1 (26.5) antibody (this panel complements Figure 6B). C) Geo MFI +/– SD of GFP expression in BEAS2B tet MR1-GFP cells pulsed with DB28 in the presence or absence of doxycycline as described in Figure 4E.

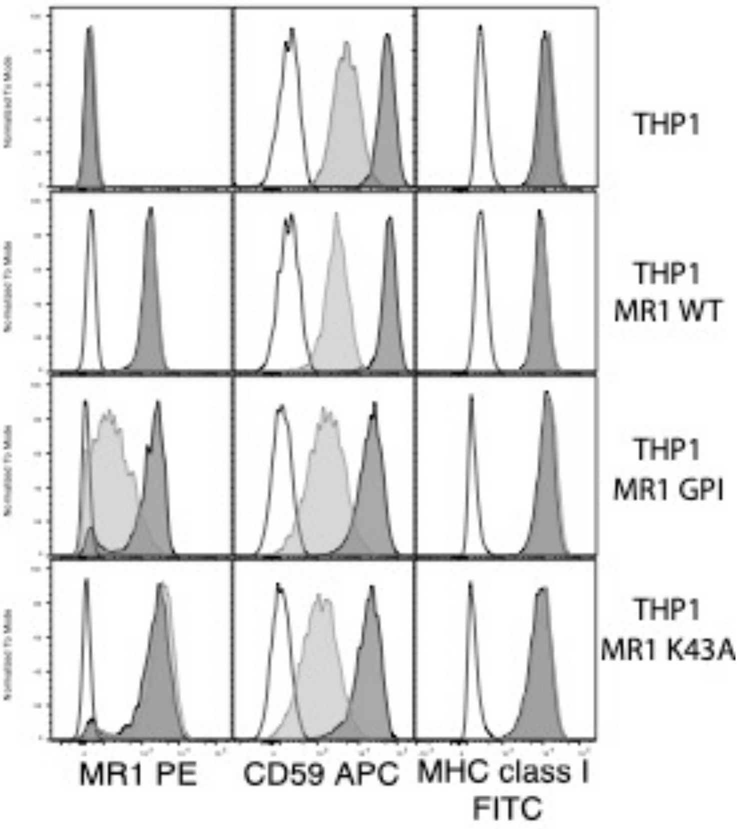

Figure S7. Profile of THP1 cell lines. THP1 cells parent line, or THP1 MR1 KO cells transduced with lentiviruses encoding for MR1 WT, MR1 GPI linked or MR1 K43A were left untreated (dark grey histograms), or were treated with PI-PLC to release GPI-linked proteins (light grey histograms). Cells were stained with antibodies to MR1, CD59 (a GPI-linked control surface protein) or MHC class I. Open histograms represent negative control staining (isotype control for PE, unstained for APC and FITC).

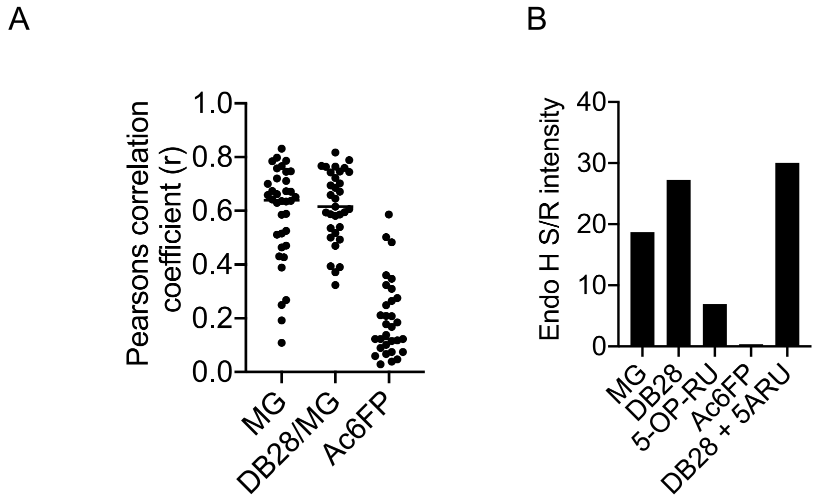

Figure S8. Confocal microscopy and immunoprecipitation quantifications. A) Pearson correlation coefficient to quantify the colocalization of MR1 with the calnexin ER marker in THP1-MR1 cells pulsed with vehicle (methylglyoxal (MG), n=36), DB28/MG (n=32) or Ac-6-FP (n=38). B) Relative amounts of EndoH-sensitive (S) and EndoH-resistant (R) MR1 molecules immunoprecipitated with mAb 26.5, following THP1 incubation with MG, DB28, 5-OP-RU, Ac-6-FP or DB28 and 5-OP-RU. This Figure complements results shown in Figure 5.

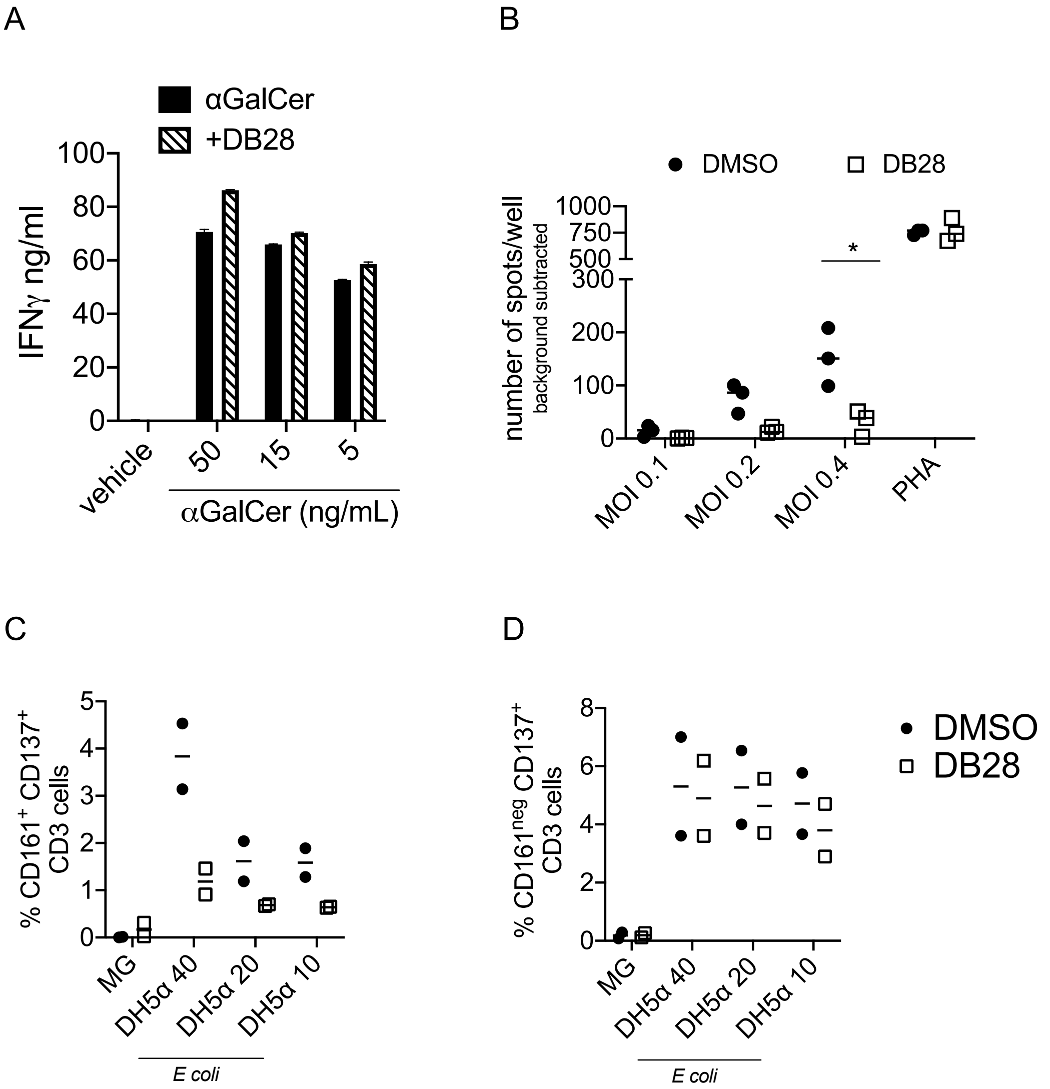

Figure S9. DB28 prevents MAIT cell activation by agonistic bacterial ligands. A) iNKT cell activation by THP1 cells pulsed with decreasing concentrations of αGalCer, in the presence or absence of DB28. Mean+/–SD of IFNγ secreted in the supernatant after 16 hours of stimulation. One experiment representative of two, each performed in technical duplicates. B) ELISpot assay for IFNγ secretion by a MAIT cell clone in response to BCG-infected BEAS2B WT cells, in the presence or absence of DB28 (20 µg/mL). Each dot represents the average of technical duplicates of one of three experiments. Average number of spots in the uninfected wells for each treatment was subtracted to normalize between experiments. Statistical significance was calculated using repeated measures ANOVA with a Bayesian Information Criterion to determine the optimal correlation structure. Tukey adjusted p value= 0.03. C and D) Activation of MAIT (C) and other CD3 cells (D) in whole blood infected with *E. coli* at the indicated MOI, in the presence or absence of DB28 (20 µg/mL). One experiment representative of three, cumulative data shown in Figure 6D.

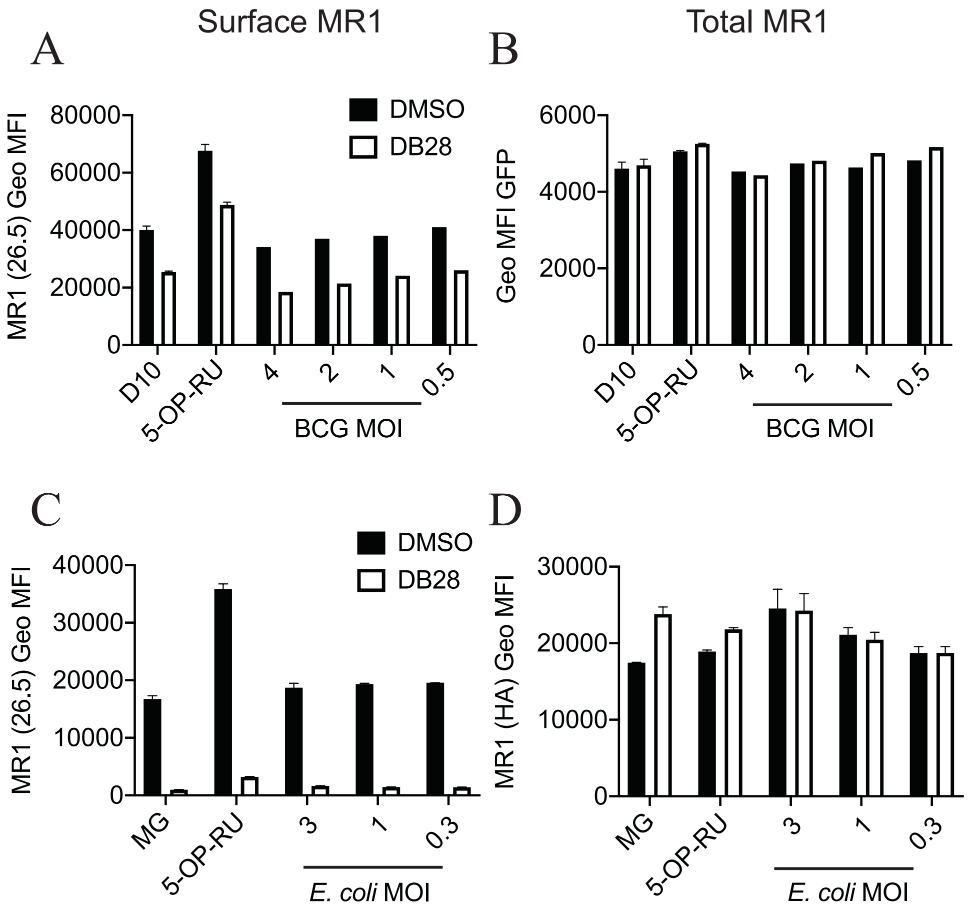

Figure S10. DB28 downregulates surface but not total MR1 in bacterial-infected cells. BEAS-2B expressing MR1-GFP (A and B) or THP1 expressing MR1-HA tagged (C and D) were infected with the indicated MOI of BCG (A and B) or *E. coli* (C and D) in the presence or absence of 20 μg/mL DB28. 5-OP-RU was used at 1 μg/mL; complete DMEM (D10) or RPMI with methyl glyoxal (MG) were used as negative controls. After overnight infection, cells were stained for MR1 cell surface expression (panels A and C, 26.5 antibody). GFP geometric mean fluorescence is plotted as proxy for total MR1 expression in BEAS2B MR1-GFP cells (panel B). Total MR1 expression determined by staining with the anti HA antibody in fixed and permeabilized THP1 MR1-HA cells is shown in panel D.

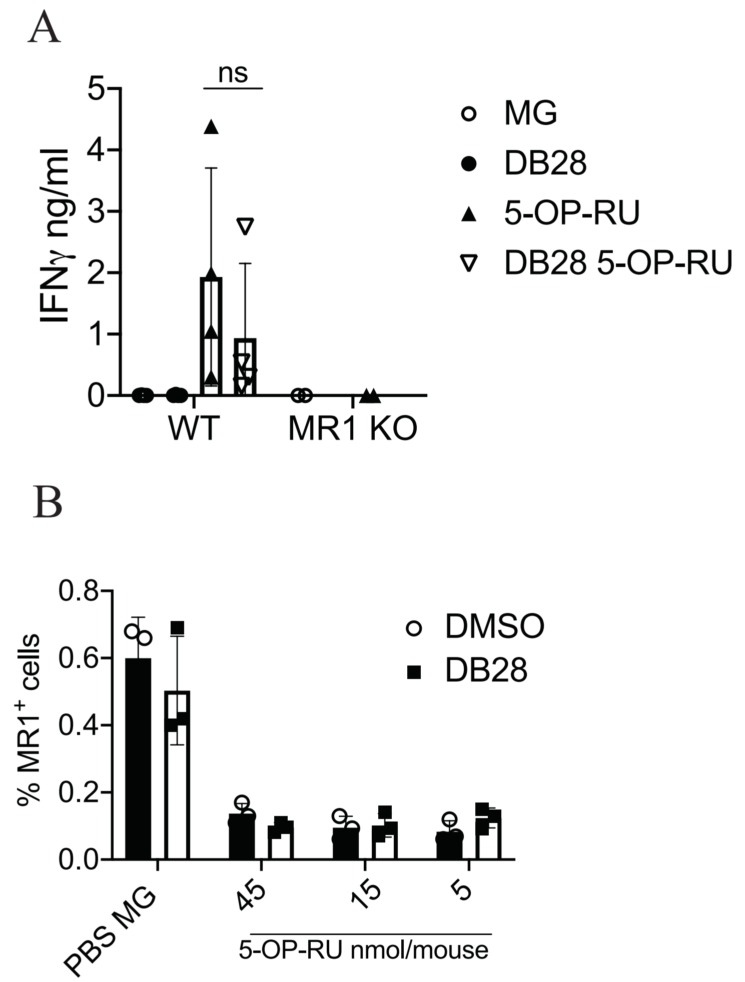

Figure S11. *In vivo* and *in vitro* activity of DB28 in mice. A) BMDC from C57BL/6 WT or MR1 KO mice were pulsed with 5-OP-RU (150 ng/mL) with or without DB28 (20 μg/mL) and incubated with human MAIT cells. Methylglyoxal (MG) was used as negative control. Plotted is the IFN-γ secretion in the supernatants at 36 hours. Cumulative data of 4 different experiments, each performed in duplicate. Two-tailed *t-test*, p 0.068 (ns).
B) C57BL/6 mice (n=3) were injected with the indicated concentration (nmol) of 5-OP-RU with or without 440 nmol DB28. The frequency of MAIT cells in the spleen was determined by MR1-tetramer staining after 24 hours. No toxicity was recorded.

References

1. Patel, O. *et al.* Recognition of vitamin B metabolites by mucosal-associated invariant T cells. *Nat. Commun.* **4**, 2142 (2013).

2. Lopez-Sagaseta, J. *et al.* MAIT recognition of a stimulatory bacterial antigen bound to MR1. *J. Immunol.* **191**, 5268-5277 (2013).

3. Talukdar, A. *et al. J. Org. Chem.* **77**, 6239-6261 (2012).

4. Roemisch, W., Eisenreich, W., Richter, G. & Bacher, A. *J. Org. Chem.* **67**, 8890-8894 (2002).

5. Philmus, B., Decamps, L., Berteau, O. & Beglev, T.P. *J. Am. Chem. Soc.* **137**, 5406-5413 (2015).

6. Cushman, M. *et al. J. Org. Chem.* **67**, 6871-6877 (2002).

7. Cushman, M., Patrick, D.A., Bacher, A. & Scheuring, J. *J. Org. Chem.* **56**, 4603-4608 (1991).

8. Cushman, M., Mavandadi, F., Kugelbrey, K. & Bacher, A. *J. Org. Chem.* **62**, 8944-8947 (1996).

9. Al-Hassan, S.S. *et al. J. Chem. So.c, Perkin Trans 1*, 2645-2656 (1980).

10. Cushman, M., Yang, D., Kis, K. & Bacher, A. *J. Org. Chem.* **66**, 8320-8327 (2001).

11. Cresswell, R.M. & H.S.C., W. *J. Chem. Soc.*, 4768-4775 (1960).

12. Corbett, A.J. *et al.* T-cell activation by transitory neo-antigens derived from distinct microbial pathways. *Nature* **509**, 361-365 (2014).

13. Eckle, S.B.G. *et al.* A molecular basis underpinning the T cell receptor heterogeneity of mucosal-associated invariant T cells. *J. Exp. Med.* **211**, 1585-1600 (2014).

14. Eckle, S.B. *et al.* A molecular basis underpinning the T cell receptor heterogeneity of mucosal-associated invariant T cells. *J. Exp. Med.* **211**, 1585-1600 (2014).

15. Keller, A.N. *et al.* Drugs and drug-like molecules can modulate the function of mucosal-associated invariant T cells. *Nat. Immunol.* **18**, 402-411 (2017).

16. Kabsch, W. XDS. *Acta Crystallogr. D* **66**, 125-132 (2010).

17. Winn, M.D. *et al.* Overview of the CCP4 suite and current developments. *Acta crystallogr. D* **67**, 235-242 (2011).

18. Adams, P.D. *et al.* PHENIX: a comprehensive Python-based system for macromolecular structure solution. *Acta Crystallogr. D* **66**, 213-221 (2010).

19. McCoy, A.J. Solving structures of protein complexes by molecular replacement with Phaser. *Acta Crystallogr. D* **63**, 32-41 (2007).

20. Emsley, P. & Cowtan, K. Coot: model-building tools for molecular graphics. *Acta Crystallogr. D* **60**, 2126-2132 (2004).

21. Chen, V.B. *et al.* MolProbity: all-atom structure validation for macromolecular crystallography. *Acta Crystallogr. D* **66**, 12-21 (2010).

22. Rahimpour, A. *et al.* Identification of phenotypically and functionally heterogeneous mouse mucosal-associated invariant T cells using MR1 tetramers. *J. Exp. Med.* **212**, 1095-1108 (2015).

23. Bedard, M. *et al.* Sterile activation of invariant natural killer T cells by ER-stressed antigen-presenting cells. *Proc Natl Acad Sci U S A* **116**, 23671-23681 (2019).
